# Regulation of plasmid-encoded isoprene metabolism in *Rhodococcus*, a representative of an important link in the global isoprene cycle

**DOI:** 10.1111/1462-2920.12793

**Published:** 2015-04-15

**Authors:** Andrew T Crombie, Myriam El Khawand, Virgil A Rhodius, Kevin A Fengler, Michael C Miller, Gregg M Whited, Terry J McGenity, J Colin Murrell

**Affiliations:** 1 University of East AngliaNorwich Research Park, Norwich, UK; 2 DuPont Industrial Biosciences925 Page Mill Road, Palo Alto, CA, 94304, USA; 3 DuPont Pioneer7200 NW 62nd Avenue, Johnston, IA, 50131, USA; 4 University of EssexWivenhoe Park, Colchester, UK

## Abstract

Emissions of biogenic volatile organic compounds (VOCs) form an important part of the global carbon cycle, comprising a significant proportion of net ecosystem productivity. They impact atmospheric chemistry and contribute directly and indirectly to greenhouse gases. Isoprene, emitted largely from plants, comprises one third of total VOCs, yet in contrast to methane, which is released in similar quantities, we know little of its biodegradation. Here, we report the genome of an isoprene degrading isolate, *Rhodococcus* sp. AD45, and, using mutagenesis shows that a plasmid-encoded soluble di-iron centre isoprene monooxygenase (IsoMO) is essential for isoprene metabolism. Using RNA sequencing (RNAseq) to analyse cells exposed to isoprene or epoxyisoprene in a substrate-switch time-course experiment, we show that transcripts from 22 contiguous genes, including those encoding IsoMO, were highly upregulated, becoming among the most abundant in the cell and comprising over 25% of the entire transcriptome. Analysis of gene transcription in the wild type and an IsoMO-disrupted mutant strain showed that epoxyisoprene, or a subsequent product of isoprene metabolism, rather than isoprene itself, was the inducing molecule. We provide a foundation of molecular data for future research on the environmental biological consumption of this important, climate-active compound.

## Introduction

Approximately one third of total volatile organic compounds (VOCs) released into the atmosphere is represented by isoprene (2-methyl-1,3-butadiene) from biological sources (400–600 Tg y^−1^), which is similar in magnitude to the methane source and comprises roughly half of total non-methane VOCs (Atkinson and Arey, 2003; Guenther *et al*., 2006; Arneth *et al*., 2008). Isoprene has an atmospheric lifetime of a few hours due to rapid photochemical degradation (Atkinson and Arey, 2003), and thus has a significant and complicated effect on global climate, reviewed by Pacifico and colleagues (2009). In the atmosphere, isoprene reacts with hydroxyl (OH) and nitrate (NO_3_) radicals and ozone (O_3_) (Atkinson and Arey, 2003). In polluted and urban environments, with high nitrogen oxide (NO_x_) levels, oxidation by OH and reaction of the resultant hydroxyperoxy radical with nitric oxide (NO) gives rise to net production of O_3_ and OH recycling. In unpolluted environments, direct reaction of isoprene with O_3_ can lead to O_3_ depletion. Globally, these reactions directly result in a net radiative forcing of 0.9 W m^−2^, with an additional effect since removal of OH radicals increases the atmospheric lifetime of methane (Pacifico *et al*., 2009). Oxidation products also form secondary organic aerosols (SOA), typified by the blue haze of the Blue Ridge Mountains of Virginia, with further implications for air quality and climate (Carlton *et al*., 2009).

Isoprene is released by plants, algae, some bacteria and animals including humans (Gelmont *et al*., 1981; Fall and Copley, 2000; Broadgate *et al*., 2004; Sharkey *et al*., 2008; Exton *et al*., 2013), usually by the action of isoprene synthase on dimethylallyl pyrophosphate (Sanadze, 2004), which, together with its isomer isopentenyl diphosphate, is produced in all organisms as an intermediate in the synthesis of essential isoprenoids (Kuzuyama and Seto, 2003). Terrestrial plants produce over 90% of the isoprene emitted to the atmosphere, with an additional contribution from marine algae (Exton *et al*., 2013). Many taxonomically diverse plants (but not all) emit isoprene, which constitutes about 2% of fixed carbon and serves to protect against thermal stress, provides protection from reactive oxygen species and in some cases acts as a signalling molecule (Loivamäki *et al*., 2008; Sharkey *et al*., 2008).

Since isoprene is an abundant natural product, it would be surprising if bacteria had not evolved to use it as a carbon and energy source, as they have evolved to use many plant-derived volatile organic compounds (reviewed by Marmulla and Harder (2014)). Indeed, oxidation of isoprene can be readily observed in soil samples (van Ginkel *et al*., 1987b; Ewers *et al*., 1990; Cleveland and Yavitt, 1997; 1998[Bibr b14],[Bibr b15]). In the 1980s and 1990s, several species of terrestrial bacteria capable of growth on isoprene were isolated (van Ginkel *et al*., 1987a,b[Bibr b25],[Bibr b26]; Ewers *et al*., 1990; Cleveland and Yavitt, 1997; van Hylckama Vlieg *et al*., 1998; Fall and Copley, 2000), but most were not characterized in detail. More recently, isoprene consumption was demonstrated in marine sediments and isolates were obtained from that environment (Acuña Alvarez *et al*., 2009). Many of the microorganisms identified in these studies were rhodococci and other Actinobacteria. The genus *Rhodococcus*, of the order Actinomycetales, comprises many aerobic non-sporulating Gram-positive bacteria abundant in soils, freshwater and marine sediments and in association with plants (Bell *et al*., 1998; Zhao *et al*., 2012). Many have impressive metabolic capabilities, possess among the largest bacterial genomes (up to 10 Mbp) and are able to transform a wide range of natural and xenobiotic compounds (Larkin *et al*., 2005), resulting in many biotechnological and industrial uses (van der Geize and Dijkhuizen, 2004).

**Figure 1:**
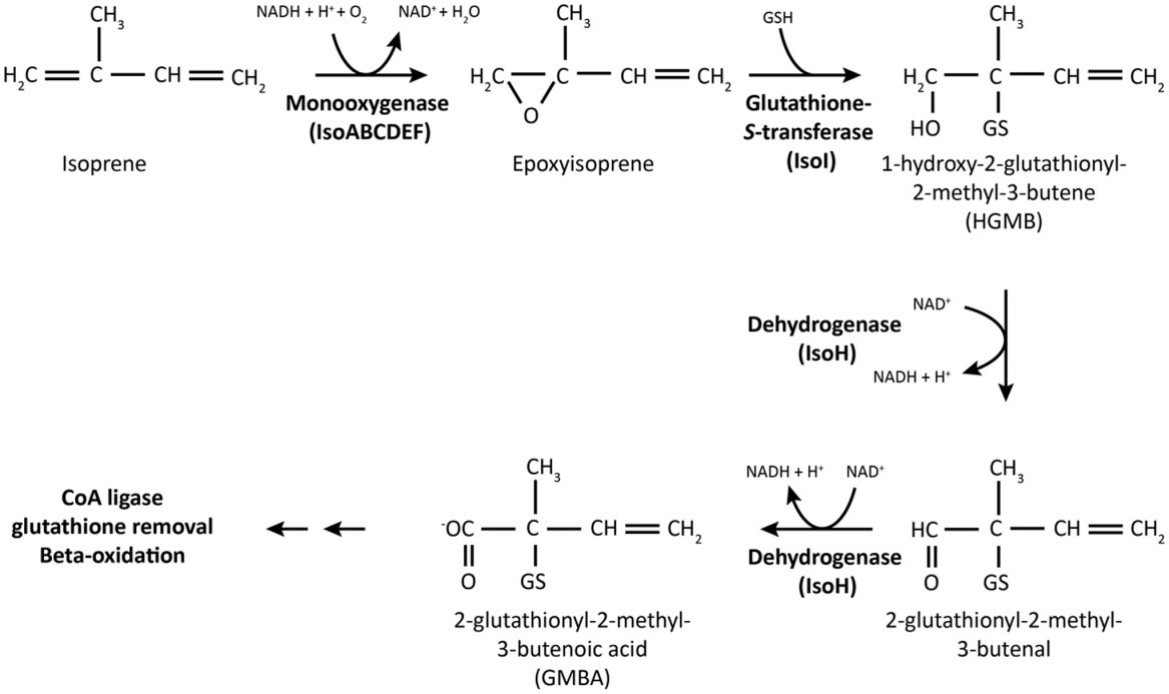
The pathway of isoprene metabolism. Re-drawn from van Hylckama Vlieg and colleagues (2000).

The most detailed characterization of an isoprene degrader was carried out 15 years ago in the lab of Dick Janssen (van Hylckama Vlieg *et al*., 1998; 1999; 2000[Bibr b32],[Bibr b33],[Bibr b34]). These workers isolated *Rhodococcus* sp. AD45 from freshwater sediment and carried out biochemical analysis of its isoprene-metabolizing ability. They identified isoprene epoxide (1,2-epoxy-2-methyl-3-butene) as the product of isoprene oxidation and purified a glutathione-*S*-transferase (IsoI) and dehydrogenase (IsoH) with activity towards isoprene epoxide and its glutathione adduct, respectively. Subsequently, they cloned and screened a *Rhodococcus* sp. AD45 gene library using a DNA probe deduced from the IsoI peptide sequence. Part of a sequence of approximately 8.5 Kbp was predicted to encode an isoprene monooxygenase (IsoMO) (*isoABCDEF)* based on proximity to *isoI* and sequence similarity to toluene monooxygenase from *Pseudomonas mendocina* KR1. Genes encoding IsoH and IsoI were identified in the region upstream (5′) of the IsoMO structural genes, which also contained two additional genes, *isoG* and *isoJ*. Although IsoJ was shown to be a glutathione-*S*-transferase, when expressed in *Escherichia coli* there was no activity towards epoxides, and no definite function in *Rhodococcus* sp. AD45 was assigned to either *isoG* or *isoJ*. The Janssen group proposed a pathway of isoprene metabolism in which the products enter central metabolism via beta-oxidation (Fig. 1). Subsequently, using polymerase chain reaction (PCR), Rui and colleagues (2004) identified additional copies of GSH-transferase genes located on the cosmid constructed in the original cloning experiments. Recent sequence analysis has shown that IsoI is only distantly related to other bacterial glutathione-*S-*transferases (Allocati *et al*., 2012). Conjugation of the epoxide with glutathione contrasts with other alkene utilizers, for example *Xanthobacter autotrophicus* PY2, *Rhodococcus rhodochrous* and *Mycobacterium* strains (Krishnakumar *et al*., 2008), which form coenzyme M conjugates with the reactive epoxides and, indeed, glutathione is relatively uncommon in Gram-positive bacteria (Allocati *et al*., 2012).

Isoprene is reactive in the atmosphere and emitted from the biosphere in amounts similar to methane, so emission rates and effects on the atmosphere have been studied in detail. Studies have also focussed on the biochemistry of isoprene production in plants, or have proposed engineering industrial isoprene biosynthesis (Whited *et al*., 2010). Despite its biological, environmental and industrial importance, the foregoing from Janssen’s group represents the sum total of our knowledge of the mechanisms of its biodegradation. The aims of this study were to improve and extend our understanding of the mechanism and regulation of isoprene metabolism, by identifying additional isoprene metabolic genes and determining their mode of regulation. We report the sequence of the *Rhodococcus* sp. AD45 genome and, using mutagenesis, show that IsoMO is essential for growth on isoprene. We identify additional genes implicated in isoprene metabolism and show that this is an inducible trait. By comparing the response of cells incubated with either isoprene or epoxyisoprene, with controls, we describe the dynamics and specifics of isoprene-responsive transcription.

## Results and discussion

### Growth and substrates of *Rhodococcus* sp. AD45

*Rhodococcus* sp. AD45 grew on succinate (specific growth rate (μ) = 0.25 ± 0.02 h^−1^ (mean ± SD), *n* = 3) or isoprene (μ = 0.16 ± 0.02 h^−1^, *n* = 6) as well as sugars (including glucose) and rich media, as sole source of carbon and energy. We found that high isoprene concentrations (> 2% v/v) inhibited growth, but using approximately 0.6% v/v, 25 ml cultures supplied with 30 μmol isoprene grew to an optical density (OD_540_) of approximately 0.5. When cells were transferred from succinate to isoprene, a lag phase of approximately 15–22 h was evident which was not present when cells were transferred from isoprene to isoprene. Many strains of *Rhodococcus* are capable of monooxygenase-mediated growth on short chain alkanes (for example propane), including *Rhodococcus erythropolis* (Kulikova and Bezborodov, 2001) or alkenes (for example propene) including *R. rhodochrous* B276 (Furuhashi *et al*., 1981). We therefore tested available strains for growth on propane, propene and isoprene, and observed that many (but not *Rhodococcus* sp. AD45) were capable of growth on propane, but that this was not correlated with growth on isoprene (Table S1).

### *Rhodococcus* sp. AD45 genome sequence

The *Rhodococcus* sp. AD45 genome is approximately 6.8 Mbp in size, with a G + C content of 61.7 mol%, and includes a 300 Kbp circular plasmid with a similar G + C content (60.6 mol%). Phylogenetic analysis based on 16S ribosomal ribonucleic acid (rRNA) gene sequences grouped *Rhodococcus* sp. AD45 within the *R. erythropolis* clade (Gürtler *et al*., 2004) (Fig. S1A). In total, 6279 protein coding sequences were predicted, including 321 on the plasmid. Table 1 shows key features of the *Rhodococcus* sp. AD45 genome in comparison with the genomes of other sequenced *Rhodococcus* strains. We identified 18 transposase sequences, of which 16 were on the plasmid.

**Table 1:** Summary of genome data from *Rhodococcus* strains

Strain	Size (Mbp)	GC (mol%)	Chr.	Plasmids	Proteins	Ref.
AD45	6.80	61.7	C	1 (C)	6279	This study
RHA1	9.70	67.0	L	3 (L)	9145	McLeod *et al*. (2006)
PD630	9.17	67.5	C	2 (C), 7 (L)	8947	Chen *et al*. (2014)
103S	5.85	68.8	C	1 (C)	4598	Letek *et al*. (2010)
PR4	6.90	62.3	C	2 (C), 1 (L)	6440	Sekine *et al*. (2006)

Strains: AD45, *Rhodococcus* sp. AD45; RHA1, *R.* sp. RHA1; PD630, *R. opacus* PD630; 103S, *R. equi* 103S; PR4, *R. erythropolis* PR4. Chr, chromosome; GC, guanine-cytosine; C, circular; L, linear.

### Alkane and alkene oxidation

Basic Local Alignment Search Tool (BLAST) searches of *Rhodococcus* genomes using, as query sequence, the α-subunit of the propane monooxygenase from propane-utilizer *Gordonia* sp. TY-5 (Kotani *et al*., 2003), revealed that many *Rhodococcus* species, including *Rhodococcus opacus* PD630, *R. rhodochrous* and *R.* RHA1 contain highly similar sequences (> 90% amino acid identity). However, we did not identify a sequence with high similarity in the genome of *Rhodococcus* sp. AD45 (the best hit was 26% identity), consistent with its inability to grow on propane. Three alkane hydroxylase *alkB* genes were identified in *Rhodococcus* sp. AD45, one of which shares 69% amino acid identity and genome context with AlkB from *Mycobacterium tuberculosis* H37Rv (Smits *et al*., 2002), which is active towards C_10_–C_16_ *n-*alkanes. Twenty-three cytochrome p450 sequences were identified, some of which may also play a role in oxidation of aromatic and aliphatic compounds (Table S2).

### Plasmid-encoded isoprene metabolic genes

**Figure 2:**
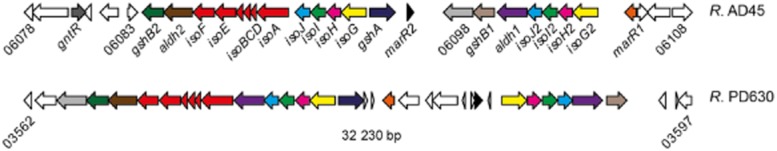
The region of the *Rhodococcus* sp. AD45 plasmid containing the isoprene metabolic genes (top) and a homologous region identified in the chromosome of *R. opacus* PD630 (bottom). The isoprene monooxygenase genes are coloured red, and other genes are colour coded according to their corresponding predicted functions.

The isoprene metabolic genes were identified on the plasmid (Fig. 2). The previously reported sequence (van Hylckama Vlieg *et al*., 2000), containing the monooxygenase (*isoABCDEF*) and four upstream genes (*isoGHIJ*) is a perfect match to nucleotides 56215–64670. Isoprene monooxygenase is a soluble diiron centre monooxygenase (SDIMO), with homology to a wide range of proteins including the soluble methane monooxygenase, propane monooxygenase, alkene monooxygenase, phenol hydroxylase and toluene monooxygenase (Notomista *et al*., 2003). These enzymes, although closely related, can be assigned to protein families based on sequence similarity and subunit arrangement, which broadly reflect substrate specificity. Phylogenetic analysis of the α-subunits (Fig. S1B) grouped IsoMO with characterized enzymes such as alkene monooxygenase (Xamo) from *Xanthobacter autotrophicus* PY2 and toluene 4-monooxygenase (T4MO) from *P. mendocina*, (α-subunit; 70% and 48% amino acid identities respectively), both of which are capable of oxidizing simple alkenes and aromatic compounds (Yen *et al*., 1991; Zhou *et al*., 1999). The monooxygenase genes *isoABCDEF* encode the hydroxylase α-subunit, hydroxylase γ-subunit, ferredoxin, coupling protein, hydroxylase β-subunit and reductase, respectively, have amino acid identities of 39–70% with the corresponding units of Xamo and are arranged in the same order (Fig. 2, Table 2). The four genes *isoGHIJ*, preceding the IsoMO genes, encode a putative coenzyme A transferase, a dehydrogenase and two glutathione transferases described previously (van Hylckama Vlieg *et al*., 1998; 1999; 2000). We identified additional copies of *isoGHIJ* in the same orientation approximately 11 kbp upstream. IsoG2, IsoH2 and IsoJ2 share 99% amino acid identity with the corresponding polypeptides encoded in the primary operon, but IsoI and IsoI2 share only 79% identity.

**Table 2:** Protein BLAST (BLASTp) hits to *Rhodococcus* sp. AD45 genes highly induced by isoprene or epoxyisoprene

Description	Gene	SZ00_	Best NCBI Blastp hit (amino acid % id)	Characterized enzyme, accession number (amino acid % id)	Organism (ref)
Hypothetical		06083	*R. wratislaviensis* NBRC 100605 (50)	–	–
Glutathione synthetase	*gshB2*	06084	*R. wratislaviensis* NBRC 100605 (81)	Glutathione synthetase, BAA22859.1 (47)	*Synechococcus* sp. PCC7942 (Okumura *et al*., 1997)
Aldehyde dehydrogenase	*aldh2*	06085	*R*. JVH1 (75)	4-hydroxymuconic semialdehyde dehydrogenase ACA50459.1 (29)	*Pseudomonas fluorescens* (Moonen *et al*., 2008)
Isoprene MO, reductase	*isoF*	06086	*R*. JVH1 (81)	Alkene MO reductase, ABS70073.1 (39)	*X. autotrophicus* PY2 (Zhou *et al*., 1999)
Isoprene MO, β-subunit	*isoE*	06087	*R*. JVH1 (84)	Alkene MO β-subunit, ABS70072.1 (52)	*X. autotrophicus* PY2 (Zhou *et al*., 1999)
Isoprene MO, coupling protein	*isoD*	06088	*R. opacus* PD630 (96)	Alkene MO coupling protein, ABS70071.1 (54)	*X. autotrophicus* PY2 (Zhou *et al*., 1999)
Isoprene MO, ferredoxin	*isoC*	06089	*R. opacus* (86)	Alkene MO ferredoxin, ABS70070.1 (48)	*X. autotrophicus* PY2 (Zhou *et al*., 1999)
Isoprene MO, γ-subunit	*isoB*	06090	*R. wratislaviensis* NBRC 100605 (85)	Alkene MO γ-subunit, ABS70069.1 (59)	*X. autotrophicus* PY2 (Zhou *et al*., 1999)
Isoprene MO, α-subunit	*isoA*	06091	*R. wratislaviensis* NBRC 100605 (92)	Alkene MO α-subunit, ABS70068.1 (70)	*X. autotrophicus* PY2 (Zhou *et al*., 1999)
Glutathione-*S*-transferase	*isoJ*	06092	*R. opacus* (90)	Disulfide-bond oxidoreductase P77526.1 (48)	*E. coli* (Wadington *et al*., 2009)
Glutathione-*S*-transferase	*isoI*	06093	*R. wratislaviensis* NBRC 100605 (87)	Failed axon connections protein, Q95RI5.1 (27)	*Drosophila melanogaster* (Hill *et al*., 1995)
Dehydrogenase	*isoH*	06094	*R*. JVH1(87)	C-factor, P21158.1 (42)	*Myxococcus xanthus* (Lee *et al*., 1995)
CoA-transferase	*isoG*	06095	*R. opacus* PD630 (91)	Succinyl-CoA:D-citramalate CoA transferase, ZP_00357883 (32)	*Chloroflexus aurantiacus* (Friedmann *et al*., 2006)
Glutamate cysteine ligase	*gshA*	06096	*R. opacus* PD630 (72)	Glutamate cysteine ligase, P9WPK7.1 (37)	*Mycobacterium tuberculosis* (Harth *et al*., 2005)
Transcriptional regulator	*marR2*	06097	*R*. JVH1 (66)	Transcriptional regulator, CAA52427.1 (30)	*Erwinia chrysanthemi* (Praillet *et al*., 1996)
CoA-disulfide reductase		06098	*R*. JVH1 (79)	CoA-disulfide reductase, P37061.1 (27)	*Enterococcus faecalis* (Ross and Claiborne, 1992)
Glutathione synthetase	*gshB1*	06099	*R*. JVH1 (88)	Glutathione synthetase, BAA22859.1 (48)	*Synechococcus* sp. PCC7942 (Okumura *et al*. 1997)
Aldehyde dehydrogenase	*aldh1*	06100	*R. opacus* (90)	Glyceraldehyde-3-phosphate dehydrogenase, EHI47090 (81)	*R. opacus* PD630 (MacEachran and Sinskey, 2013)
Glutathione-*S*-transferase	*isoJ2*	06101	*R. opacus* (91)	Disulfide-bond oxidoreductase P77526.1 (48)	*E. coli* (Wadington *et al*. 2009)
Glutathione-*S*-transferase	*isoI2*	06102	*R*. *JVH1* (88)	Failed axon connections protein, Q95RI5.1 (27)	*Drosophila melanogaster* (Hill *et al*., 1995)
Dehydrogenase	*isoH2*	06103	*R*. JVH1 (88)	C-factor, P21158.1 (42)	*Myxococcus xanthus* (Lee *et al*., 1995)
CoA-transferase	*isoG2*	06104	*R. opacus* PD630 (91)	Succinyl-CoA:D-citramalate CoA transferase, ZP_00357883 (32)	*Chloroflexus aurantiacus* (Friedmann *et al*., 2006

MO, monooxygenase.

Five predicted open reading frames (ORFs) separate these duplicated genes. Immediately upstream of *isoG*, and divergently transcribed, *gshA* encodes glutamate-cysteine ligase, the first enzyme of glutathione biosynthesis. An additional copy of *gshA* is located on the *Rhodococcus* sp. AD45 chromosome (SZ00_04638), with approximately 37% amino acid identity to the plasmid-encoded copy. Downstream (3′) of *isoJ2* and transcribed in the same direction, genes encode an aldehyde dehydrogenase (*aldh1*), glutathione synthetase (*gshB1*) and predicted coenzyme A-disulfide reductase (SZ00_06098). Ahead of *gshA* is a *marR*-type transcriptional regulator (*marR2*), although not in this case arranged in the typical orientation, i.e. divergently transcribed from its regulatory target (Alekshun and Levy, 1999).

Downstream of the monooxygenase genes (*isoABCDEF*), a second putative aldehyde dehydrogenase, *aldh2*, is located, very similar to sequences from *R.* JVH1, *R. opacus* PD630 and *R. wratislaviensis* NBRC 100605 (73–75% amino acid identity). Apart from these three, highly similar sequences were not found in other strains, for example *R.* RHA1 or *R. erythropolis* CCM2595 nor in the National Center for Biotechnology Information (NCBI) database (max 30% identity). A second copy of glutathione synthetase, *gshB2*, (67% amino acid identity with GshB1) follows *aldh2*. The subsequent ORF (SZ00_06083), in the opposite orientation, is predicted to encode a protein (157 amino acids) of unknown function with no conserved domains. This protein shares 55–59% identity with sequences from *R.* JVH1, *R. opacus* PD630 and *R. wratislaviensis* NBRC 100605, and around 35% identity with numerous other *Rhodococcus* strains. A predicted phytanoyl-CoA dioxygenase, hypothetical protein and *gntR*-type transcriptional regulator (229 amino acids with 54% identity to a sequence from *Pseudonocardia* sp. P1), are adjacent. A second *marR*-type regulator (*marR1*) is predicted at the other end of the cluster, ahead of *isoG2*. Although sequences with high similarity to most of these genes are present in the databases, ascribed functions are mostly not based directly on experimental evidence. Table 2 lists these predicted proteins and characterized examples.

### Key genes for isoprene degradation

To verify that IsoMO was essential for isoprene metabolism, we constructed a deletion mutant of *Rhodococcus* sp. AD45 in which 435 bp of the *isoA* coding sequence was replaced by an antibiotic resistance cassette. This mutant strain grew on succinate and nutrient broth similarly to the wild type, but showed no growth whatsoever on isoprene. Based on transcriptional data (see later), we predicted that not only the monooxygenase but also several additional genes were important in isoprene utilization. As shown previously (van Hylckama Vlieg *et al*., 1998; 1999[Bibr b32],[Bibr b33]), glutathione is involved in isoprene metabolism in *Rhodococcus* sp. AD45, and we found that the genomes of other isoprene degraders isolated in our lab from several environments contained *isoI*-like glutathione-*S*-transferase genes in the vicinity of the IsoMO genes (M. el Khawand, in preparation, A. Johnston, in preparation). We searched available sequenced genomes for high similarity homologues of *isoA* and *isoI*, in close proximity, and identified *R.* JVH1, *R. wratislaviensis* NBRC 100605 and *R. opacus* PD630 as potential isoprene degraders. Since there is a published complete genome sequence for *R. opacus* PD630 (Chen *et al*., 2014), the strain was tested and grew on isoprene, which has not, to our knowledge, been previously reported. The isoprene-cluster genes in *Rhodococcus* sp. AD45 and *R. opacus* PD630 are compared in Fig. 2. In contrast to their location on a plasmid in *Rhodococcus* sp. AD45, in *R. opacus* PD630 they are on the chromosome. All other genes predicted to be unique to isoprene utilization in *Rhodococcus* sp. AD45 are present in *R. opacus* PD630 (amino acid identity 50–90%) and in a similar layout, except that duplicated genes *isoG2H2I2J2* are in the opposite orientation and separated from their homologues by additional genes not present on the *Rhodococcus* sp. AD45 plasmid. Also, an additional copy of *aldh1* is located between *isoA* and *isoI* in *R. opacus* PD630.

### Expression of the isoprene metabolic genes is inducible

**Figure 3:**
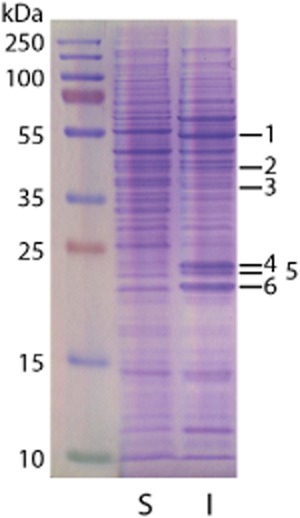
Polypeptide profiles of soluble extract from cells grown to late-exponential phase on succinate (S) or isoprene (I), separated by SDS-PAGE. The bands indicated were cut from the lane loaded with isoprene extract and identified by mass spectrometry. Identifications of the polypeptides from the isoprene cluster are shown in Table 3, together with the number of peptides used for identification and the theoretical molecular mass of the polypeptide.

To determine if isoprene oxidation in *Rhodococcus* sp. AD45 was an inducible trait, we examined the activity and soluble protein profiles of cells grown on succinate or isoprene. Cells grown on succinate did not possess hexene epoxidation activity (Fig. S2A), nor contain large amounts of the IsoMO and associated polypeptides (Fig. 3), in contrast to isoprene-grown cells. We also noted that growth of cultures supplied with succinate alone was indistinguishable from cultures supplied with succinate plus isoprene, up to the point at which the succinate-only incubations reached stationary phase, after which the succinate-plus-isoprene cultures continued growing at a reduced rate. There was also no reduction of isoprene in these vials until this point, suggesting that isoprene was not consumed until succinate was depleted, (Fig. S2B). These data suggest that isoprene metabolic genes were induced by isoprene but that uptake was repressed by the presence of a preferred carbon source (succinate).

**Table 3:** Mass-spectrometric identifications of bands cut from the gel shown in Fig. 3

Band	Identification	Peptides	kDa
1	IsoA	9	49.7
2	IsoE	12	38.5
3	IsoF	6	37.3
	GshB2	6	39.1
4	IsoJ/IsoJ2	9	26.3
	IsoI	6	27.1
5	IsoI2	16	26.9
	IsoI	8	27.1
	IsoJ/IsoJ2	7	26.3
6	IsoH/IsoH2	13	24.0

### General metabolic potential of *Rhodococcus* sp. AD45

We searched the *Rhodococcus* sp. AD45 genome, guided in part by the metabolic abilities of other *Rhodococcus* strains. We identified putative genes for DNA replication and partitioning, central carbon metabolism, biosynthesis of storage compounds and aromatic compound degradation (Fig. S3 and Table S2). Genes required for two mechanisms of propionate and fatty acid metabolism were identified, as reported in *Mycobacterium* (Savvi *et al*., 2008). Notable was the absence of genes encoding Entner–Doudoroff pathway enzymes 6-phosphogluconate dehydratase and 2-keto-3-deoxy-6-phosphogluconate aldolase, polyhydroxyalkanoate biosynthesis and phenylacetate degradation, although these are present in other rhodococci (Navarro-Llorens *et al*., 2005; McLeod *et al*., 2006; Alvarez *et al*., 2013; Chen *et al*., 2014). Genes required for conjugative transfer, such as *traA* from *R. erythropolis* AN12 (Yang *et al*., 2007) or transfer genes found in *R. erythropolis* PR4 (Sekine *et al*., 2006), were not found, although the plasmid encodes a putative relaxase (SZ00_06343), functionally related to TraA.

### Transcriptome analysis by RNAseq

To examine isoprene-related gene expression, we conducted a replicated time-course experiment in which succinate-grown *Rhodococcus* sp. AD45 cells were starved and then exposed to isoprene, or the product of isoprene oxidation, epoxyisoprene. The onset of isoprene- or epoxyisoprene-induced gene expression was evaluated by sequencing the transcriptome, in comparison with controls either exposed to succinate (the original growth substrate), glucose or incubated without any additions (no-substrate), over time. Samples were removed at time-point zero (T0) (immediately prior to substrate addition) and at T1–T5, corresponding to 19, 43, 75, 240 min and 25 h. A total of 475 million reads were generated resulting in a target sequencing depth of three to five million reads per sample (minimum two million reads), sufficient for robust detection of many differentially expressed genes in replicated studies with bacteria (Haas *et al*., 2012). For expression analysis, reads were mapped to predicted coding sequences (CDS), quantified as reads per kilobase per million mapped reads (RPKM), and hence assigned to one of 7 arbitrarily defined expression levels (Table S3).

Since at T0, immediately prior to substrate addition, all samples had received identical treatment, we used data from 15 biological replicates to provide a robust picture of T0 transcription. At this time-point mean transcript abundance for each gene varied between 1 and 47 500 RPKM. Two thirds of genes were transcribed in the range 12.5–312.5 RPKM, 8% were not transcribed (< 2.5 RPKM) and 0.07% were very highly transcribed (> 7,812.5 RPKM). Next, we examined expression of ‘housekeeping’ genes *rpoB*, *gyrA* and *gmk*, which encode core cellular functions, not specific to any particular substrate. For each gene, the experimental conditions induced changes in expression within an approximately fourfold range (Fig. S4A), which, with few exceptions, followed similar trends. We used these data as an indication of the minimum factor required to identify specific substrate-induced differential gene expression.

### Transcription of isoprene metabolic genes

**Figure 4:**
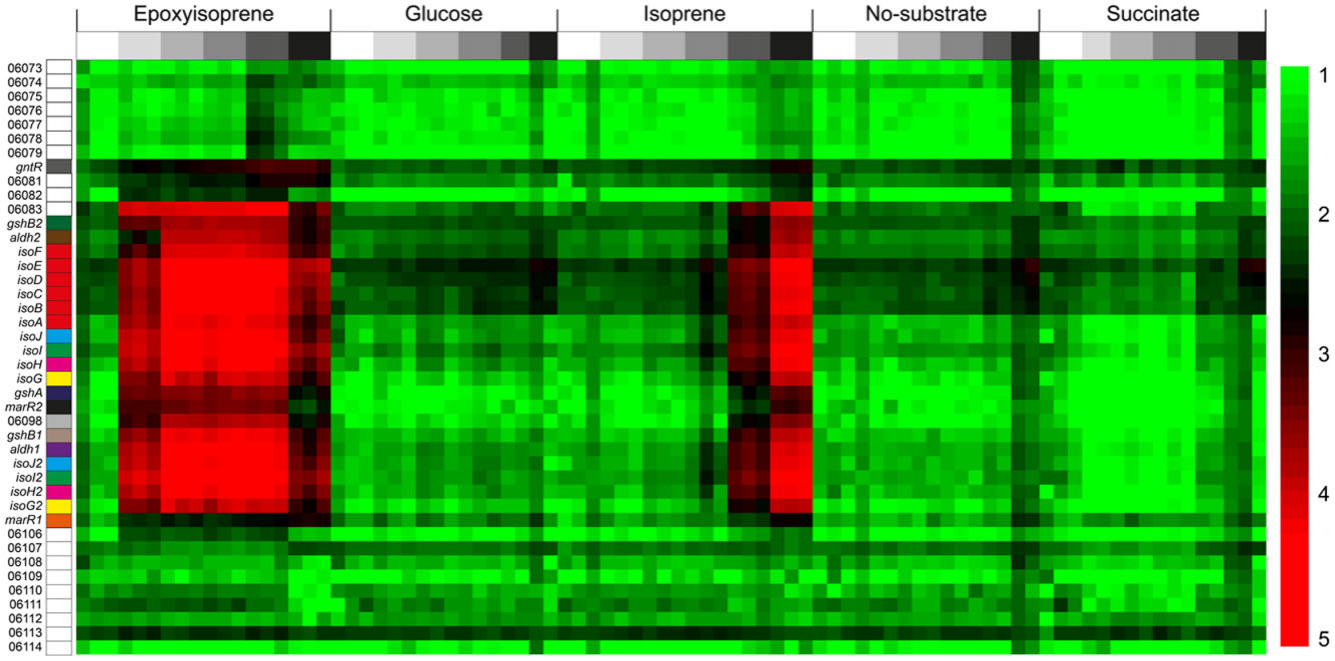
Induction of isoprene-responsive gene transcription. Normalized transcript abundance (RPKM) of 42 genes (vertical axis) from the *Rhodococcus* sp. AD45 plasmid, centred on the isoprene-responsive cluster and colour coded as Fig. 2. The samples (84) (horizontal axis) were induced with the substrates shown. Time points are indicated with shading (above), from T0 (white) to T5 (black). The scale bar on the right shows log_10_ RPKM. Transcripts of 22 genes, SZ00_06104–SZ00_06083, averaged 17–211 RPKM at T0 (mean of 15 replicates), increasing to maxima of over 24 000 (mean of *isoI*) when induced by isoprene at T5, or over 35 000 (mean of *isoE*) when induced by epoxyisoprene at T3.

**Figure 5:**
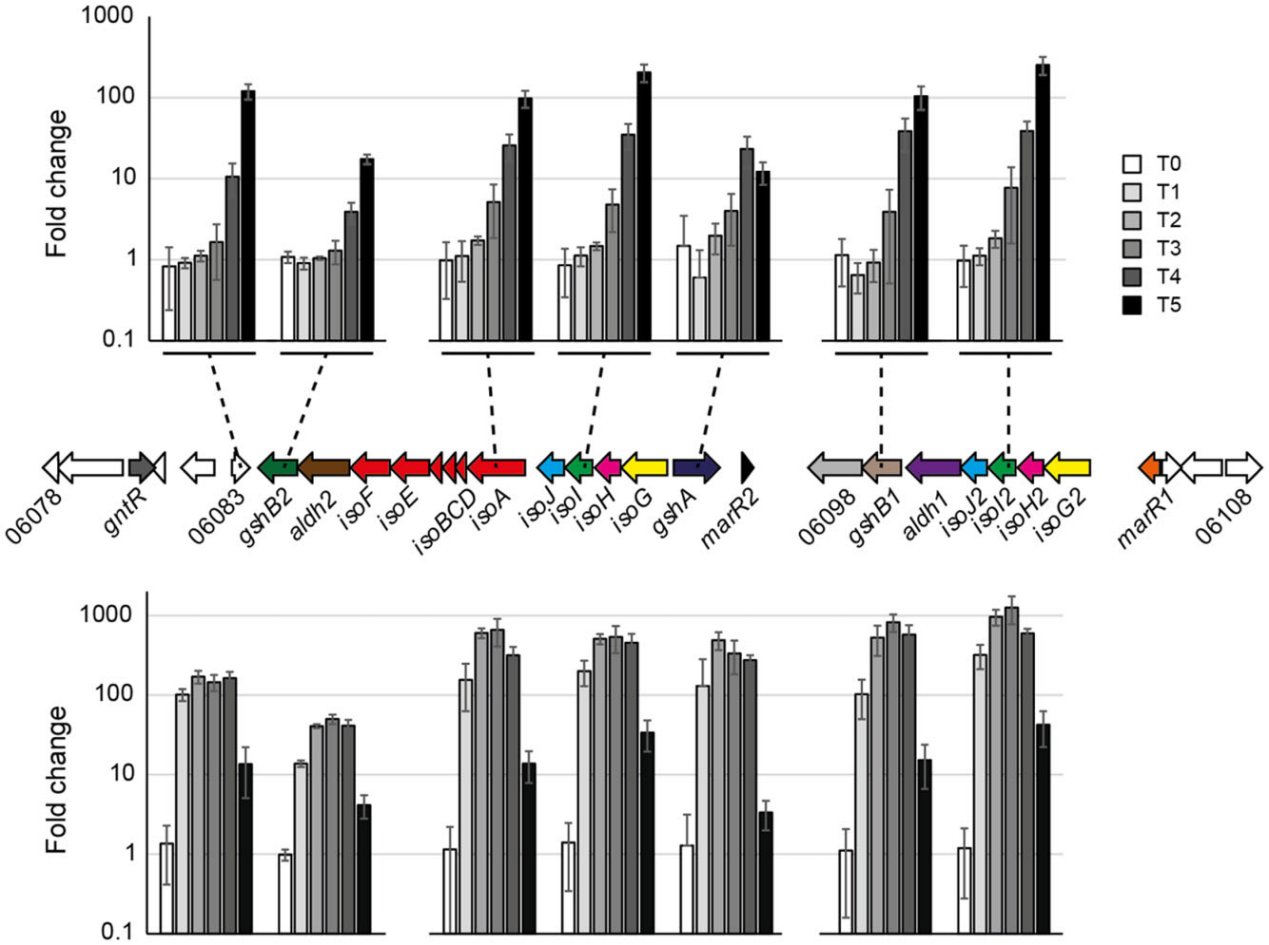
Upper bar chart: transcript upregulation of seven representative genes out of 22 from the *Rhodococcus* sp. AD45 isoprene cluster (as indicated) showing the increase in relative abundance (RPKM) from T0 to T5 in isoprene-induced samples. Lower bar chart: as above, except samples induced by epoxyisoprene. All data show a comparison with no-substrate controls at the same time points. Data show the mean ± SD of three replicates, except T0, 15 replicates, no-substrate T4 and T5, two replicates each. The charts show the extremely high level of transcript induction in cells exposed to both isoprene and epoxyisoprene, with an even more rapid response to epoxyisoprene, with close to maximum transcript levels already reached by T2 (43 min).

Since the genome contained nearly identical copies of *isoG*, *isoH* and *isoJ*, only a small proportion of reads could be uniquely assigned to one or other copy (2–27% for isoprene T5 samples), so expression of these duplicates was considered together. However, due to their lower comparative similarity, most of the reads aligning to *isoI* and *isoI2* could be uniquely assigned. In samples with the highest levels of isoprene-induced transcripts, expression levels of reads unique to both copies of all four genes were extremely similar, suggesting that both groups of duplicates were in fact transcribed at similar levels. At T0, transcripts corresponding to the 22 genes involved in isoprene conversion (SZ00_06104–SZ00_06083), including *isoGHIJABCDEF*, were detected at moderate levels ranging from 17–211 RPKM (Fig. 4) (mean transcript levels for 15 T0 replicates, relative standard deviation (RSD) between 10–100% depending on gene). Control samples exposed to no-substrate or succinate showed an average threefold or twofold (respectively) increase in these transcripts between the start and end of the experiment. In contrast, when exposed to isoprene, transcription increased dramatically from T3 (75 min) until the end of the experiment (12–254-fold increase at T5), compared with non-induced (no-substrate) controls at the same time points (Figs 4 and 5). Furthermore, incubation with the first product of isoprene oxidation, epoxyisoprene, resulted in a higher and even more rapid induction of the isoprene-responsive genes, reaching a maximum (up to 1000-fold) at T3, before declining by the end of the experiment, presumably due to depletion of the inducing substrate (Fig. 5). Thus, both isoprene and epoxyisoprene induced a high level of transcription of 22 genes, which became among the most abundant transcripts in the cell, together comprising over 25% of the entire transcriptome. A genome-wide search did not reveal any isoprene-responsive genes that did not also respond to epoxyisoprene (see *Experimental procedures* for details).

### Inducers of isoprene metabolism

**Figure 6:**
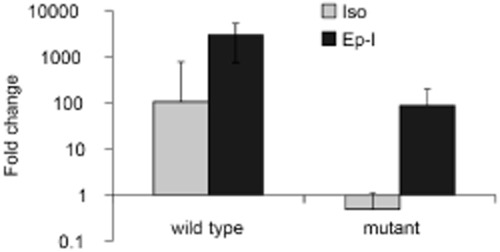
The effect of *isoA* deletion on isoprene-responsive transcription. Quantitative reverse transcription polymerase chain reaction data showing transcription of *isoG* at 3.75 h in wild-type and *isoA-*deletion mutant cells exposed to isoprene (Iso) or epoxyisoprene (Ep-I). Data show the mean of three biological replicates ± SD, relative to *rpoB* transcripts and are normalized to time zero, before the addition of substrate.

Since epoxyisoprene also induced expression of all genes induced by isoprene, it seemed likely that epoxyisoprene (or a subsequent metabolite), rather than isoprene itself, was the inducing molecule, although we could not discount the possibility that isoprene was also an inducer, albeit slower or less effective. Since the *isoA* deletion strain was unable to oxidize isoprene and could not form the potential inducer, epoxyisoprene, from isoprene, we used quantitative reverse transcription PCR (RT-qPCR) to examine transcription of *isoG* in the *isoA* deletion strain during incubations with isoprene or epoxyisoprene. There was no induction of *isoG* transcripts during incubations of this strain with isoprene, whereas cells incubated with epoxyisoprene showed a 100-fold increase at 3.75 h after addition of substrate (Fig. 6), demonstrating that these cells did not respond to isoprene as inducer.

### Additional genes induced during isoprene metabolism

We searched for any other genes responsive to either isoprene or epoxyisoprene, in addition to the isoprene-responsive cluster highlighted in Fig. 4. Transcripts of a further 26 genes were more abundant (4–110 fold) at one or more of time points T1, T2 and T3 in epoxyisoprene-induced cells compared with T0 (Fig. S4B), but none of these was also more abundant in isoprene-induced cells (at T5) compared with succinate or non-induced (no-substrate) cells at the same time points (fourfold cut-off). These data suggest that only the 22 genes identified in Fig. 4 were specifically required for isoprene metabolism, and that these additional genes were transcribed in response to stimuli such as starvation or toxicity stress. Genes of central metabolism, expected to be required for growth on isoprene but not necessarily for growth on succinate or glucose, for example isocitrate lyase, malate synthase and the methylmalonyl-CoA or methylcitrate pathway-encoding genes, required for assimilation of acetyl-CoA and propionyl-CoA, were not transcribed differentially between the different treatments nor was the chromosomally encoded *gshA*.

Of the three transcriptional regulators located in the vicinity of the isoprene metabolic genes, *marR1* did not show an isoprene-responsive change in transcription level across the experiment. In contrast, *marR2* transcripts were upregulated 19-fold in isoprene-induced compared with uninduced cells at T5, and showed the same progressive increase over time as the isoprene metabolic genes (Fig. S4C). The *gntR* regulator, at the end of the cluster, remained stable between T0 and T4 but was threefold more abundant at the final time point in isoprene-induced cells.

### Co-transcribed genes and transcription boundaries

Since isoprene-related transcripts were highly abundant in induced cells, we were able to analyse transcriptional boundaries of these genes by mapping reads to the entire sequence rather than to CDS as used for expression analysis. Reads were then visualized using Integrative Genome Viewer (IGV) (Thorvaldsdóttir *et al*., 2013). Although read abundance decreased gradually over the region *isoGHIJABCDEF*, we did not detect any start or termination sites here, except at the start of the cluster, ahead of *isoG* (Fig. S5A). In particular, transcription termination or initiation was not found in the *isoJ-isoA* intergene region (326 bp) which contains a possible transcriptional terminator identified previously (van Hylckama Vlieg *et al*., 2000). This finding was verified by RT-PCR and Rapid Amplification of cDNA Ends (5′-RACE), which confirmed transcripts spanning this region and did not identify a transcriptional start site (Fig. S5B). However, a rapid increase in read abundance, denoting a transcription start site, was evident approximately 68 bp 5′ of the *isoG* and *isoG2* start codons, a finding confirmed by 5′-RACE, which identified the same start site (Fig. S5C). Both copies of *isoG* share an identical nucleotide sequence extending 73 nucleotides upstream from the predicted initiation codon, indicating similar relative transcription start sites for both copies. The lack of transcripts in the region separating divergently transcribed genes *gshA* and *isoG* also pointed to a transcription start for the former in this region. An increase of transcript reads in the region ahead of hypothetical protein-encoding gene SZ00_06083 suggested that this gene was transcribed as a single unit. Putative promoter sequences could also be detected in advance of these transcriptional start sites (not shown).

## Conclusions

In order to better understand the mechanism and role of isoprene-degrading microorganisms, here we present the genome of an isoprene-degrading *Rhodococcus* strain, the first published complete sequence of an isolate known to degrade this environmentally important compound. In comparison with other *Rhodococcus* strains, for example RHA1, strain AD45 has a reduced genome size and more specialized metabolic potential. Genes for isoprene metabolism were concentrated in a small region on a megaplasmid, containing a relatively large number of transposase sequences, suggesting the possibility of horizontal transfer of plasmid-encoded genes. The later stages of the isoprene metabolic pathway have not been biochemically characterized, but a hypothesis was proposed by van Hylckama Vlieg and colleagues (2000). These authors showed that isoprene was oxidized to the epoxide, conjugated with glutathione, and subject to two dehydrogenation steps, catalysed by *isoABCDEF, isoI* and *isoH* respectively (Fig. 1). They proposed conversion of the product of these reactions, 2-glutathionlyl -2-methyl-3-butenoic acid, to the coenzyme A thioester, followed by removal of the glutathione moiety, possibly catalysed by IsoG and IsoJ. This product could plausibly be broken down into acetyl CoA and propionyl CoA, possibly sharing enzymes with the latter part of the isoleucine degradation pathway (Massey *et al*., 1976).

In this study, we used transcriptional analysis of cells exposed to a substrate switch to identify previously unknown genes required for isoprene metabolism. By examining the changes in gene expression induced by exposure of succinate-grown cells to isoprene or epoxyisoprene, we aimed to identify sequences transcribed by the cell as it synthesizes the cellular machinery required for isoprene metabolism, culminating, at the final time point, with the expression of proteins needed for growth on isoprene. Although the high levels of transcripts during adaptation to new conditions may not be maintained during steady state, this approach is extremely sensitive in identifying differentially expressed transcripts required for the altered metabolic conditions. Analyses showed a high or extremely high level of transcription of 22 contiguous genes when induced by isoprene or epoxyisoprene, strongly suggesting that all are involved in isoprene metabolism. Most have a readily predictable function, including the monooxygenase, glutathione transferase, dehydrogenase (IsoH) and glutathione biosynthesis genes. In addition, genes annotated as encoding two aldehyde dehydrogenases, a disulfide reductase and hypothetical protein were highly induced by isoprene. While we can be confident that these are involved in isoprene metabolism, their exact functions remain to be determined. Genome wide, no other genes showed a high level of upregulation in response to isoprene, suggesting that this cluster may contain all the genes specific to its metabolism. These were not induced by isoprene in a strain unable to oxidize isoprene to epoxyisoprene, demonstrating that a subsequent metabolite and not isoprene itself was the inducing molecule. The data strongly suggest that *isoGHIJABCDEF* were co-transcribed as an operon, with a promoter upstream of *isoG*. As part of an investigation into the molecular regulation of isoprene metabolism, the three transcriptional regulators located in the cluster are the subject of continued study in our laboratory, as are the latter stages of the isoprene metabolic pathway. In this study, we have identified the complete set of inducible genes responsible for isoprene degradation. These findings have implications for biogeochemical cycling of isoprene, considerably advance our understanding of isoprene metabolism and provide the foundations for continuing studies of isoprene degradation in the environment.

## Experimental procedures

*Rhodococcus* sp. AD45 was a gift from Dick Janssen, University of Groningen, the Netherlands. *Rhodococcus opacus* PD630 was obtained from the Deutsche Sammlung von Mikroorganismen und Zellkulturen culture collection. *Rhodococcus* strains were grown on minimal medium as described (van Hylckama Vlieg *et al*., 1998), with isoprene or succinate (5 mM) or on nutrient broth (0.8% w/v). Isoprene was added as a gas to the headspace of culture vials by addition of 1/100 volume of vapour removed from a small vial containing a small quantity of liquid isoprene, heated to 37°C in a water bath, resulting in a concentration in the headspace of culture vials of approximately 0.6% (v/v). Headspace isoprene was accurately quantified by injection of 100 μl of headspace gas into an Agilent 7890A gas chromatograph fitted with an HP-Plot/Q column (30 m, 530 μm bore, 40 μm film) at an oven temperature of 175°C, injector 250°C (1:5 split ratio) and flame ionization detector at 300°C (carrier gas He, 4 ml min^−1^), and comparison with standards containing a known quantity of isoprene in air. For growth on plates, media were solidified with Bacto agar (1.5% w/v). Antibiotics for *Rhodococcus* sp. AD45 were used at a concentration of 100 μg ml^−1^ (kanamycin) or 5 μg ml^−1^ (gentamicin).

### Epoxidation assay

Epoxide-forming ability was assayed by a modification of the epoxide assay previously described (Cheung *et al*., 2013). Hexene was used as substrate since the epoxyhexane product of IsoMO oxidation is an irreversible inhibitor of epoxide degradation in *Rhodococcus* sp. AD45 (van Hylckama Vlieg *et al*., 1998). Cell suspensions (3–30 mg dry weight re-suspended in 200 μl phosphate buffer 50 mM, pH 7.0) were incubated with 2 μl hexene in sealed 2 ml vials for 1 h at 30°C. 4-(4-nitrobenzyl)pyridine (NBP) (400 μl of 100 mM in ethylene glycol) was added and vials incubated at 80°C for 30 min. Vials were cooled and 500 μl of triethylamine/acetone (1:1) was added. Development of a blue colour indicated formation of epoxide (epoxyhexane).

### Molecular methods

Deoxyribonucleic acid was extracted from mid-late exponential cultures using a previously described method (Asturias and Timmis, 1993). Deoxyribonucleic acid manipulations were performed using standard methods (Sambrook *et al*., 2001).

### Genome sequencing and assembly

#### Library preparation and sequencing

Approximately 1 μg of high-quality *Rhodococcus* sp. AD45 genomic DNA was prepared for sequencing using the Nextera Mate Pair Sample Preparation Kit (Illumina, catalog #: FC-132–1001). A gel-free mate pair library was prepared following the manufacturer’s instructions, which typically yields mate pair fragments with a peak distribution between 2 to 4 Kbp, and an overall wide distribution of 1 Kbp to 15 Kbp. Briefly, a tagmentation reaction was performed to simultaneously fragment the DNA and tag the ends with a biotinylated ‘junction’ adapter. A polymerase was then used in a strand displacement reaction to make the adapter–fragment junctions flush. Fragments were then purified, self-ligated and any remaining non-circular DNA eliminated by exonuclease digestion. The DNA junction adapter self-ligated circles were sheared by Covaris sonication to approximately 400 bp, and fragments containing the biotinylated junction adapters attached to the two original tagmentation ends were captured using streptavidin magnetic beads, repaired, A-tailed and ligated to indexed TruSeq adapters. The library was finally PCR amplified, clustered and sequenced on an Illumina MiSeq Desktop sequencer according to the manufacturer’s protocols. Sequencing was performed at both ends of clustered DNA fragments using paired-end sequencing primers for both Read1 and Read2 (Illumina). The resulting read 1 and read 2 sequences were grouped into ‘read pairs’ according to the X and Y coordinates of the corresponding DNA cluster on the flow cell. Sequencing reads and quality scores were generated in a real-time fashion with the Illumina Data Collection Software rta 1.17. After initial base calling, additional custom filtering was performed using calibrated quality scores generated by the Illumina pipeline. Reads generated from both ends of DNA fragments were trimmed by removing from the 3′ ends bases with a Phred-equivalent quality score below 10. A length threshold of 24 was applied to filtering, indicating that all bases < 24 bases in length after trimming were removed from further analysis.

#### Genome assembly and annotation

Nextera junction adapter sequences were trimmed from the reads using cutadapt (http://www.code.google.com/p/cutadapt/) resulting in a sequence dataset of 9 287 414 reads (7 454 088 paired and 1 833 326 single end). The sequences were assembled into contigs using the high-quality mate-pair option in SPAdes version 3.1.0 (Bankevich *et al*., 2012) resulting in 10 contigs (> 500 bp). To evaluate the assembly, reads were re-aligned to the contigs and visually inspected with IGV. The assembly was manually curated to break up misassemblies, correct single nucleotide polymorphisms and insertion/deletion errors, and to merge contigs. Ribosomal repeat regions that could not be fully resolved were broken off into separate consensus contigs. The final draft assembly is composed of nine contigs including one putative plasmid, one 16S ribosomal repeat contig and one 23S ribosomal repeat contig with a total assembly size of 6 794 789 bp. Genome annotation was performed with Prokka (Seemann, 2014).

### RNA-seq

#### Sample preparation and sequencing

*Rhodococcus* sp. AD45 was grown in nine 2 L conical flasks each containing 400 ml of minimal medium with succinate (20 mM) as carbon source, using an inoculum (10 ml each) from a late-exponential succinate-grown culture. Cells were harvested after 16 h at mid-late exponential phase, centrifuged (5000 *g*, 24°C, 20 min), washed twice in minimal medium without substrate and combined into three replicate cell suspensions of 110 ml minimal medium without substrate, each of which was then divided among five 250 ml flasks. These were starved by incubation at 30°C with shaking for 1 h before addition of either glucose, succinate (both 10 mM final concentration), epoxyisoprene (2.5 mM) or isoprene (approximately 0.6% (v/v)). The no-substrate controls did not receive any carbon source. Immediately before addition of the substrate, and subsequently at 19, 43, 75, 240 min and 25 h (designated T0–T5), four 0.5 ml aliquots of cells were removed from each flask, immediately treated with RNAprotect Bacteria Reagent (Qiagen catalogue #76506) following the manufacturer’s instructions, and stored at -80° C prior to analysis. Ribonucleic acid was extracted using a QIAGEN RNeasy 96 kit (QIAGEN; catalogue #74181) following the manufacturer’s instructions. Using 2 μg of total RNA for each sample, rRNA was removed using the RiboZero rRNA Removal Kit (Meta-Bacteria) (Epicentre; catalogue #RZMN11086), and the final RNA samples were purified using Beckman Coulter RNAClean XP magnetic beads. Using the TruSeq RNA Sample Preparation Kit v2 (Illumina; catalogue #RS-122–2001), the RNA was chemically sheared and complementary (c)DNA primed using random hexamers to generate first and second strand cDNA fragments ranging from 50 bp to 500 bp (average 180 bp). The cDNA ends were filled in, 3′ adenylated and synthesized and adapters were ligated. Twenty-four Illumina indexes were used for deconvolution, and the samples were sequenced on an Illumina HiSeq2500, 12 samples per lane, generating 50 bp + 6 bp index reads. Images from the sequencing runs were analysed via the Illumina analysis pipeline and the resulting sequences filtered for quality: bases with Q scores of less than 20 were trimmed, and any resultant sequence reads less than 24 bp were removed. The reads were then split into samples by their index identifier.

#### Read alignment and quantification

The following samples were removed due to failed sequencing reactions: succinate T1 replicate 1, glucose T3 replicate 1, no substrate T4 replicate 3 and no substrate T5 replicate 3. The remaining sample reads were aligned to the *Rhodococcus* sp. AD45 genome sequence or just the coding sequences using Bowtie2 in the Genedata Refiner Genome software package (http://www.genedata.com) with the settings ‘End-to-End Alignment’, ‘Sensitive’ and either ‘Best Alignment’ for standard quantification of gene expression or ‘All Alignments’ to enable multiple alignments and quantification over duplicated genomic regions such as the isoprene operon genes. For identification and quantification of reads mapping uniquely to high-similarity duplicate genes, a mapping quality (MQ) filter was applied to remove all reads with MQ < 5 from the analysis. From all 86 samples, 571 047 049 reads aligned to the genome and 130 908 410 reads aligned to the coding sequences using the ‘Best Alignment’ setting. Each sample contained at least two million reads that aligned to the genome.

Reads aligned to the coding sequences were quantified using Genedata Refiner Genome. The quantified expression matrices were normalized to relative parts per kilobase per million (RPKM) and analysed using the package Genedata Analyst. Sample expression reproducibility was addressed using a cross-correlation matrix of the RPKM values for all samples: succinate T5 replicate 2 and glucose T5 replicate 2 were removed due to poor sample replicate correlation (*R* < 0.8). Thus 80% of samples (including all isoprene-induced and epoxyisoprene-induced) contained three biological replicates, and all samples included at least two. At T0, relative standard deviation of transcript RPKM levels of 15 replicates varied between 6% and 316% (mean 55%) for each gene, decreasing for genes with higher transcription levels (mean 17% above 312.5 RPKM).

To identify any genes responsive to isoprene but not to epoxyisoprene, we compared samples showing the maximum levels of isoprene-related transcripts, i.e. T5 for isoprene-induced and T3 for epoxyisoprene-induced samples. However, since there was an inevitable effect on the transcriptome related to the sampling time point, to identify transcriptional changes that were substrate- rather than time point-related, we discounted genes that were not also more abundant compared with succinate-induced or no-substrate-induced (control) samples at the same time points, and also those that were not more abundant compared with T0, using a fourfold cut-off. Similarly, to identify all genes induced by isoprene, we searched for transcripts more abundant in both isoprene (T5) and epoxyisoprene (T3) compared with T0, and which were also upregulated compared with succinate or no-substrate-induced cells at the same time points, using the same fourfold cut-off.

### Validation by RT-qPCR

We used RT-qPCR to validate the RNAseq data in cells exposed to isoprene. Initially, to determine suitable time points for the RNAseq analyses, we carried out a preliminary investigation by determining *isoA* transcripts in comparison with *rpoB* (encoding the β-subunit of RNA polymerase) as a stable reference. Subsequently, using one of the three isoprene replicates generated in the RNAseq experiment, we quantified both *isoA* and *isoG* in comparison to *rpoB*. Both these independent experiments confirmed the RNAseq data. RNAseq analysis showed *isoA* and *isoG* upregulated by 222-fold and 385-fold, respectively, at 25 h compared with T0. As determined by RT-qPCR, *isoA* transcripts were 182-fold higher at 18 h (first experiment) or 151-fold higher at 25 h (second experiment) than at T0 (Fig. S4D). Transcripts of *isoG* were 884-fold higher at 25 h, although since our PCR primers did not distinguish between the two *isoG* copies, this figure represents the sum of the transcripts of both genes.

### RT-PCR, RACE and RT-qPCR

Ribonucleic acid for RT-qPCR was extracted using a hot-phenol method previously described (Gilbert *et al*., 2000) or using a lipid tissue kit (Qiagen) in conjunction with RNeasy kit (Qiagen). For the latter method, cell pellets were re-suspended in 100 μl TE buffer containing lysozyme (15 mg ml^−1^), mixed by vortexing and incubated at room temperature for 10 min with vortexing every few minutes. Following the addition of 1 ml hot (65°C) Qiazol reagent, tubes were vortexed (3 min) and incubated for 5 min at room temperature. The mixture was transferred to Lysing Matrix B tubes (MP Biomedicals) and shaken at setting 6 for 30 s in a FastPrep bead beating machine (MP Biomedicals). The supernatant was extracted with 200 μl chloroform : isoamylalcohol (24:1) and centrifuged (12 000 *g*, 15 min, 4°C) and the supernatant transferred to fresh tubes. Ethanol (500 μl) was added and the RNA purified using an RNeasy kit (Qiagen) following the manufacturer’s instructions. For both RNA extraction methods, residual DNA was removed with two off-column treatments with RNase-free DNase (Qiagen) following the manufacturer’s instructions. Ribonucleic acid concentration was measured using a NanoDrop spectrophotometer (Thermo Fisher) and quality checked by agarose gel electrophoresis or using an Experion system (BioRad) following the manufacturer’s instructions. Polymerase chain reaction using 16S rRNA primers was used to check for DNA contamination. Complementary DNA was synthesized using Superscript II or Superscipt III (Invitrogen) reverse transcriptase following the manufacturer’s instructions, using 100–650 ng of total RNA and priming with random hexamers (Fermentas), including negative controls in which reverse transcriptase was omitted from reactions. Polymerase chain reaction across the *isoJ-isoA* inter-gene region used primers isoJA_F (5′-CGATTGCCGATATCTCAACC-3′)/isoJA_R (5′-GATCGACGTAGCTTAGATCC-3′). 5′ RACE was carried out using a Roche Next Generation 5′ RACE kit, following the manufacturer’s instructions, using gene-specific primers isoA_GSP1 (5′-ACTGCCTTGACGCCCGATTC-3′), isoA_GSP2 (5′-ACGTAATCGCGGTACGAGAC-3′) and isoA_GSP3 (5′-GGAAGGCCTCAGATGGATCG-3′) (for *isoA*), or isoG_GSP1 (5′-CCCGACATCATCGAACACAG-3′), isoG_GSP2 (5′-TCGGGCCGCTCATGGATAAC-3′) and isoG_GSP3 (5′-AACGCCTTTCCTCTTGCTG-3′) (for *isoG*). Quantitative PCR was conducted in 20 μl reactions using a StepOnePlus instrument (Applied Biosystems) using FastSYBR green master mix, primers (250 nM) designed to amplify 63–100 bp and 2 μl template. Complementary DNA was quantified against standards prepared from serial dilutions of cDNA synthesized from isoprene-grown cells, which were included in every plate. For qPCR, primers were gmk_qF (5′-TGAGGTGGACGGCAAGGA-3′)/gmk_qR (5′-GAATCGATCATCCGCTGAAAC-3′), gyrA_qF (5′-TTTCTTGTCGTACTGAATGGTGAGTA-3′)/gyrA_qR (5′-CGCCACTTCCGGTGGTTAC-3′), isoA_qF (5′-CGCAGAAAGCTCTCGATATCG-3′)/isoA_qR (5′-CGGACCGGTTAACGTCTGAA-3′), isoG_qF (5′-AGGGTGCGGATGTCATCAAG-3′)/isoG_qR (5′-TTCGGCAGTGAACGAACATG-3′) and rpoB_qF (5′-GCATCCCCGAGTCGTTCA-3′)/rpoB_qR (5′-GAGGACAGCACCTCCACGTT-3′).

### Mutagenesis of *isoA*

An *isoA* deletion strain was constructed by marker exchange mutagenesis as described previously (Schäfer *et al*., 1994). Briefly, approximately 500 bp was amplified by PCR using primers MekAF (5′- AATGGAAGGCGCAGATAATG-3′)/MekAR (5′- GCATAAGCTTTTGAGCAGGTCATGGGAGA-3′) and MekBF (5′- GCATAAGCTTGTGGATCGTCAATCATCACG-3′)/MekBR (5′- GCGGTCGATAATGTTCTGGT-3′) from regions of the genome of *Rhodococcus* sp. AD45 at each end of the *isoA* coding sequence. These were cloned into pK18mobsacB^8^, and a gentamicin cassette, excised from p34S Gm (Dennis and Zylstra, 1998), inserted into the EcoRI site. This construct was introduced into *Rhodococcus* sp. AD45 cells by electroporation. To prepare cells for electroporation, 50 ml cultures were grown in minimal medium with succinate to mid-exponential phase, cooled on ice, harvested by centrifugation (2500 *g*, 15 min, 4°C), washed twice in ice-cold water and re-suspended in 1 ml 10% (w/v) glycerol. Electroporation conditions were 2.5 kV, 800 Ω, 25 μF using 100 μl of cell suspension in a 2 mm cuvette. Cells were recovered for 4 h in 1 ml minimal medium with shaking at 30°C before plating on selective media containing gentamicin. A second recombination event and removal of the vector backbone were subsequently facilitated by spreading cells on plates containing sucrose (10% w/v) and screening for sensitivity to kanamycin and resistance to gentamicin. The intended gene deletion was checked by PCR using primers 3723F (5′-ATTCTCGGGACGCGAATGTG-3′)/5296R (5′-AGGAAGGCGAGGCCAAGTAG-3′), located outside of the cloned regions, and sequencing.

### Blast searches of *Rhodococcus* genomes

A nucleotide database was constructed from published *Rhodococcus* genomes and queried with amino acid sequences using local tblastn in BioEdit.

### SDS-PAGE

*Rhodococcus* sp. AD45 cells grown on succinate or isoprene, for the proteomic analysis shown in Fig. 3, were broken by three passages through a French pressure cell (American Instrument) at 110 MPa (on ice). Cell debris was removed by centrifugation (10 000 *g*, 15 min, 4°C). Proteins were separated by SDS-PAGE, and bands of interest were excised from the gel for the identification of polypeptides by the Biological Mass Spectrometry and Proteomics Facility in the School of Life Sciences, University of Warwick, UK. Coomassie Brilliant Blue-stained gel pieces were processed and tryptically digested using the manufacturer’s recommended protocol on the MassPrep robotic protein handling system (Micromass, Manchester, UK). The extracted peptides from each sample were analysed by nano liquid-chromatography electrospray-ionization tandem mass spectrometry (LC-ESI-MS/MS) using NanoAcquity/Q-ToF Ultima Global instrumentation (Waters Corporation, Manchester, UK) with a 15 min liquid chromatography gradient. All MS and MS/MS data were corrected for mass drift using reference data collected from human [Glu^1^]-fibrinopeptide B (catalogue F3261, Sigma). The data were used to interrogate a database compiled from predicted coding sequences of *Rhodococcus* sp. AD45 using the Waters ProteinLynx Global Server v2.5.1.

### Accession number

This Whole Genome Shotgun project has been deposited at DDBJ/EMBL/GenBank under the accession JYOP00000000. The version described in this paper is version JYOP01000000.

## Supporting Information

**Fig. S1.** (A) 16S rRNA gene-based phylogenetic relationship between *Rhodococcus* sp. AD45 (shown boxed) and other representative strains. The tree, drawn using the neighbour-joining method, was constructed using mega6 (Tamura *et al*., 2013). All positions containing gaps and missing data were eliminated. There were a total of 1321 nucleotide positions in the final dataset. Bootstrap values (1000 replications) greater than 50% are shown at the nodes. The scale bar shows nucleotide substitutions per site. (B) Un-rooted phylogenetic tree based on amino acid sequences, relating the *Rhodococcus* sp. AD45 isoprene monooxygenase hydroxylase α-subunit with other representative enzymes. The tree was constructed using the maximum likelihood method in mega6 (Tamura *et al*., 2013). All positions containing gaps and missing data were eliminated. There were a total of 481 positions in the final dataset. Bootstrap values (500 replications) greater than 75% are shown at the nodes. The scale bar shows amino acid substitutions per site. **Fig. S2.** (A) Epoxide forming ability of cells grown on isoprene (cuvettes 1, 3, 6) or succinate (cuvettes 2, 4) when incubated with hexene. The positive control, containing cells incubated with epoxyhexane, is shown in cuvette 5. Blue colouration indicates the presence of epoxide. **B**) Growth of *Rhodococcus* sp. AD45 on succinate or succinate plus isoprene. Isoprene headspace concentration (when grown on succinate plus isoprene) is shown on the secondary (right) axis. Data show the mean ± SD of three replicates. **Fig. S3.** Replication region of *Rhodococcus* sp. AD45. (A) The genome in the region of the putative replication origin, showing DnaA box and *parS* sequences. The consensus sequences from *Bacillus subtilis* (Moriya *et al*., 1985) and Gram-positive bacteria (Livny *et al*., 2007) are shown. (B) Guanine-cytosine (GC) asymmetry calculated using the web-based server GenSkew (http://www.genskew.csb.univie.ac.at/). **Fig. S4.** (A) Transcripts of *rpoB*, *gyrA* and *gmk*, encoding the β-subunit of RNA polymerase, the α-subunit of DNA gyrase and guanylate kinase, respectively, compared over five treatments and six time points. The bar chart shows expression level (transcript abundance, RPKM), normalized to the mean level across all conditions for each gene. Data show the mean of three replicates ± SD except for six samples that were excluded as described in *Experimental procedures*. T0–T5 refer to the sampling time points (time zero to 25 h) as described in the text. (B) Upregulation of genes, additional to the isoprene cluster SZ00_06080–SZ00_06106, in epoxyisoprene-induced cells at time points T1, T2, T3, compared with T0. All genes upregulated by at least fourfold in any of time points T1–T3, compared with T0, are shown. Data show the mean fold-change of three replicates ± SD. SZ00_05719–SZ00_05730 show homology to sequences possibly involved in amino alcohol metabolism (Nagy *et al*., 1995). SZ00_04855–SZ00_04859 comprise a putative enterobactin exporter, peroxidase, multidrug resistance protein and hypothetical proteins and SZ00_04607–SZ00_04609 are annotated as copper transport proteins. None of these genes, however, was also upregulated (> 4-fold) in comparison with non-induced (no-substrate) and succinate-induced samples at the same time points. (C) Upregulation of three regulatory genes from the isoprene cluster showing the change in abundance (fold-change) from T0 to T5 in isoprene-induced samples in comparison with no-substrate controls at the same time points. Data show the mean of three replicates ± SD, except T0, 15 replicates. (D) Upregulation of *isoA* (left) and *isoA* and *isoG* (right), in succinate grown cells, induced by isoprene, quantified by RT-qPCR. The data are normalized to *rpoB* as reference gene, expressed relative to time-point zero, prior to induction, and show the mean ± SD of three technical replicates from the two independent experiments, quantifying *isoA* (left) or both *isoA* and *isoG* (right). **Fig. S5.** (A) Transcript reads mapped to the isoprene cluster region of the *Rhodococcus* sp. AD45 genome, displayed in IGV (Thorvaldsdottir *et al*., 2013). The trace, indicating read abundance, shows replicate 2 induced with isoprene at T5, and is representative of three replicates. The y-axis shows read coverage at each nucleotide position. The ORFs are shown below. The trace indicates that transcription start sites are located upstream of *isoG, gshA* and SZ00_06083. Additionally, no termination of transcription is apparent in the *isoJ – isoA* intergene space. (B) PCR using primers spanning the *isoJ*-*isoA* intergene region using a cDNA template. Complementary DNA was synthesized from RNA extracted from cells grown on succinate (lane 1), isoprene (3) or epoxyisoprene (5). Lane 7 used a DNA template. Lanes 2, 4, 6 omitted reverse transcriptase from the cDNA synthesis reactions. Lane 8, no template control. Expected product size, 617 bp. The amplification of cDNA from all three growth conditions demonstrates the continuity of transcription across this region. (C) 5′ RACE was used with RNA extracted from cells grown on epoxyisoprene (lane 1), isoprene (2) or succinate (3). The 125 bp band was cloned and sequenced, indicating a transcriptional start site approximately 75 bp 5′ of the *isoG* start codon. **Table S1.** Growth of laboratory strains on propene, propane and isoprene. **Table S2.** List of *Rhodococcus* sp. AD45 genes referred to in the text. **Table S3.** Frequency distribution of gene transcription. Transcript abundance is shown as per cent of total RPKM for each sample and time point. Data show the mean of three replicates or 15 replicates (T0) except that six samples in total were excluded as described in *Experimental procedures*.
