# Supplementary material for: Regulation of plasmid-encoded isoprene metabolism in Rhodococcus, a representative of an important link in the global isoprene cycle
Source: Environ Microbiol. 2015 Apr 15;17(9):3314–29. doi: 10.1111/1462-2920.12793 (PMC4676930; doi:10.1111/1462-2920.12793)
Supplement: Supplementary file 1 [file emi0017-3314-sd1.pdf]

Supplementary Information

Supplementary Tables

Table S1. Growth of laboratory strains on propene, propane and isoprene

Strain	Propene	Propane	Isoprene
Rhodococcus sp. AD45	-	-	+
R. opacus PD630	-	+	+
R. opacus DSM 1069	**n.d.**	+	-
X. autotrophicus PY2	+	**n.d.**	-
R. rhodochrous B276	+	+	-
R. rhodochrous PNKb1	-	+	-
R. aetherivorans I24	**n.d.**	+	-
Rhodococcus sp. RHA1	-	+	-

n.d., not determined.

Table S2. List of *Rhodococcus* sp. AD45 genes referred to in the text

Central metabolism

Enzyme	Gene	EC.	SZ00_	Best NCBI Blastp hit	Amino acid % id	Pathway/function	Comments (reference)
Phosphoglycerate mutase	*gpmA*	5.4.2.11	00076	*R.* sp. P27	243/248 (98%)	Glycolysis	
Isocitrate lyase	*aceA*	4.1.3.1	00123	*R. erythropolis* PR4	429/429 (100%)	Glyoxylate bypass	
Dihydrolipoyl dehydrogenase	*lpdA*	1.8.1.4	00150	*R. erythropolis* PR4	454/467 (97%)	Central metabolism	
Acetate kinase	*ackA*	2.7.2.1	00224	*R. qingshengii*	362/388 (93%)	Acetate metabolism	
Phosphate acetyltransferase	*pta*	2.3.1.8	00225	*R. erythropolis* PR4	626/692 (90%)	Acetate metabolism	
Fructose bisphosphate aldolase	*fba*	4.1.2.13	00291	*R. qingshengii*	330/345 (96%)	Pentose phosphate	
Phosphoenolpyruvate carboxykinase	*pck*	4.1.1.32	00724	*R.* sp. P27	599/608 (99%)	Gluconeogenesis	
Succinate dehydrogenase s/u	*sdhC*	1.3.5.1	01383	*R. erythropolis*	215/248 (87%)	TCA cycle	
Succinate dehydrogenase s/u	*sdhA*	1.3.5.1	01384	*R.* sp. P27	605/649 (93%)	TCA cycle	
Succinate dehydrogenase s/u	*sdhB*	1.3.5.1	01385	*R. erythropolis*	236/249 (95%)	TCA cycle	
Methylmalonyl-CoA epimerase	*mce*	5.1.99.1	02324	*R. erythropolis*	150/160 (94%)	Methylmalonyl-CoA pathway	
Ribose-5-phosphate isomerase B	*rpiB*	5.3.1.6	02411	*R. erythropolis* PR4	154/157 (98%)	Pentose phosphate	
Ribokinase	*rbsK*	2.7.1.15	02448	*R. qingshengii*	230/287 (80%)	Pentose phosphate	
Pyruvate dehydrogenase comp E1	*aceE*	1.2.4.1	02558	*R. erythropolis* PR4	905/951 (95%)	Central metabolism	
6-phosphofructokinase	*pfkA*	2.7.1.11	02669	*R. erythropolis* PR4	338/343 (99%)	Glycolysis/pentose phosphate	
Pyruvate carboxylase	*pyc*	6.4.1.1	02713	*R. erythropolis* PR4	1099/1134 (97%)	Central metabolism	
Malate:quinone oxidoreductase	*mqo*	1.1.5.4	02819	*R. erythropolis* SK121	491/512 (96%)	TCA cycle	
Phosphocarrier protein	*ptsH*		02976	*R. erythropolis* PR4	79/85 (93%)	Sugar metabolism	
PTS transporter subunit	*fruA*		02977	*R. qingshengii*	623/694 (90%)	Sugar metabolism	
Phosphofructokinase	*pfk*	2.7.1.11	02978	*R. erythropolis* PR4	258/310 (83%)	Glycolysis/sugar metabolism	
Sugar metabolism regulator	*glcR*		02979	*R. qingshengii*	232/255 (91%)	Sugar metabolism	
Phosphotransferase PTS enzyme 1	*pts1*	2.7.3.9	02980	*R. erythropolis* SK121	480/545 (88%)	Sugar metabolism	
Glucokinase	*ppgK*	2.7.1.63	03027	*R. erythropolis* CCM2595	215/244 (88%)	Central metabolism	
Sugar transporter	*xylE*		03047	*R. qingshengii*	449/480 (94%)	Sugar metabolism	
Malate dehydrogenase	*mdh*	1.1.1.37	03158	*R. erythropolis* PR4	289/328 (88%)	TCA cycle	
Dihydrolipoyl transacetylase	*pdhC*	2.3.1.12	03267	*R.* JVH1	260/379 (69%)	Central metabolism	
Phosphoenolpyruvate synthase	*ppsA*	2.7.9.2	03306	*R. erythropolis*	612/752 (81%)	Gluconeogenesis	
Ribulose-phosphate 3-epimerase	*rpe*	5.1.3.1	03374	*R. erythropolis* PR4	214/227 (94%)	Pentose phosphate	

Glyceraldehyde-3-phosphate dehydrogenase	*gapA*	1.2.1.12	03384	*R. erythropolis* PR4	337/339 (99%)	Glycolysis	
Phosphoglycerate kinase	*pgk*	2.7.2.3	03385	*R. erythropolis* SK121	399/403 (99%)	Glycolysis	
Triosephosphate isomerase	*tpi*	5.3.1.1	03386	*R. erythropolis* PR4	252/261 (97%)	Glycolysis /pentose phosphate	
Phosphoenolpyruvate carboxylase	*ppc*	4.1.1.31	03389	*R. qingshengii*	856/926 (92%)	Central metabolism	53% id to CAA32450.1 from *C.* glutamicum (1)
6-phosphoglucolactonase	*pgl*	3.1.1.31	03412	*R. qingshengii*	226/246 (92%)	Pentose phosphate	
Glucose-6-phosphate dehydrogenase s/u	*opcA*		03413	*R. erythropolis* SK121	290/302 (96%)	Pentose phosphate	
Glucose-6-phosphate 1-dehydrogenase	*zwf*	1.1.1.49	03414	*R. erythropolis* SK121	489/514 (95%)	Pentose phosphate	
Transaldolase	*tal*	2.2.1.2	03415	*R. qingshengii*	370/377 (98%)	Pentose phosphate	
Transketolase	*tkt*	2.2.1.1	03416	*R. erythropolis* SK121	686/700 (98%)	Pentose phosphate	
Aconitate hydratase	*acnA*	4.2.1.3	03443	*R. erythropolis*	921/933 (99%)	TCA cycle	
Methylmalonyl-CoA mutase s/u	*mcmA*	5.4.99.2	03468	*R. erythropolis* CCM2595	589/631 (93%)	Methylmalonyl-CoA pathway	
Methylmalonyl-CoA mutase s/u	*mcmB*	5.4.99.2	03469	*R. erythropolis* SK121	735/742 (99%)	Methylmalonyl-CoA pathway	
6-phosphogluconate dehydrogenase	*ygjI*	1.1.1.44	03482	*R. erythropolis*	484/485 (99%)	Pentose phosphate	
Gluconolactonase	*pgl*	3.1.1.17	03572	*R. triatomae*	261/312 (84%)	Pentose phosphate	
Malate synthase	*aceB*	2.3.3.9	03691	*R. erythropolis* CCM2595	710/727 (98%)	Glyoxylate bypass	
Dihydrolipoyl transacetylase	*pdhC*	2.3.1.12	04014	*R. qingshengii*	418/437 (96%)	Central metabolism	
2-methylcitrate dehydratase	*prpD*	4.2.1.79	04293	*R. erythropolis*	472/500 (94%)	Methylcitrate cycle	
2-methylisocitrate lyase	*prpB*	4.1.3.30	04294	*R. erythropolis* SK121	295/305 (97%)	Methylcitrate cycle	
2-methylcitrate synthase	*prpC*	2.3.3.5	04295	*R. erythropolis*	355/373 (95%)	Methylcitrate cycle	
Pyruvate carboxylase	*pyc*	6.4.1.1	04296	*R. erythropolis* CCM2595	1024/1136 (90%)	Gluconeogenesis	67% id with *pyc* from *C.* glutamicum (2)
Citrate synthase	*gltA*	2.3.3.1	04401	*R. erythropolis* PR4	370/375 (99%)	TCA cycle	
Citrate synthase type II	*gltA*	2.3.3.1	04405	*R. erythropolis* PR4	424/432 (98%)	TCA cycle	
Glycerol kinase	*glpK*	2.7.1.30	04412	*R.* erythropolis SK121	489/502 (97%)	Central metabolism	
Glucose-6-phosphate isomerase	*pgi*	5.3.1.9	04532	*R. erythropolis* PR4	504/553 (91%)	Pentose phosphate	
Succinyl-CoA synthetase β-subunit	*sucC*	6.2.1.5	04537	*R. erythropolis* PR4	388/389 (99%)	TCA cycle	
Succinyl-CoA synthetase α-subunit	*sucD*	6.2.1.5	04538	*R. erythropolis* PR4	296/300 (99%)	TCA cycle	
Ribose-phosphate pyrophosphokinase	*prs*	2.7.6.1	04645	*R. triatomae*	307/326 (94%)	Central metabolism	
Enolase	*eno*	4.2.1.11	04673	*R. erythropolis* PR4	426/428 (99%)	Glycolysis	
Serine hydroxymethyltransferase	*glyA*	2.1.2.1	04728	*R. erythropolis* PR4	425/435 (98%)	One-carbon metabolism	
Fumarate hydratase	*fum*	4.2.1.2	04735	*R. erythropolis* PR4	448/467 (96%)	TCA cycle	

Fructose 1-6-bisphosphatase	*glpX*	3.1.3.11	04736	*R. erythropolis* PR4	343/344 (99%)	Gluconeogenesis
Malate dehydrogenase (oxaloacetate-decarboxylating) (NADP+)	*maeB*	1.1.1.40	04835	*R. erythropolis* PR4	381/397 (96%)	Central metabolism
2-oxoglutarate dehydrogenase	*sucA*	1.2.4.2	04845	*R.* sp. P27	1201/1257 (96%)	TCA cycle
Pyruvate kinase	*pyk*	2.7.1.40	04986	*R. erythropolis* CCM2595	428/444 (96%)	Glycolysis
Pyruvate dehydrogenase (quinone)	*poxB*	1.2.5.1	05156	*R. erythropolis* CCM2595	543/591 (92%)	Central metabolism
Glucokinase	*glk*	2.7.1.2	05209	*R. erythropolis*	281/319 (88%)	Glycolysis
Dihydrolipoamide succinyltransferase	*sucB*	2.3.1.61	05245	*R. qingshengii*	500/584 (86%)	TCA cycle
Phosphoglucomutase	*pgmA*	5.4.2.2	05338	*R. erythropolis*	513/545 (94%)	Glycolysis/pentose phosphate
Isocitrate dehydrogenase (NADP+)	*icd*	1.1.1.42	05558	*R. erythropolis* PR4	403/407 (99%)	TCA cycle (NADP-dependent)
Propionyl-CoA carboxylase α-subunit	*pccA*	6.4.1.3	05779	*R. erythropolis*	652/661 (99%)	Methylmalonyl-CoA pathway
Propionyl-CoA carboxylase β-subunit	*pccB*	6.4.1.3	05780	*R. erythropolis* PR4	532/532 (100%)	Methylmalonyl-CoA pathway
PTS glucose transporter subunit IIA	*ptsG*		05917	*R. erythropolis* CCM2595	134/149 (90%)	Sugar metabolism

Storage compounds

Enzyme	gene	SZ00_	Best NCBI Blastp hit	Amino acid % id	Pathway/function	Comments (reference)
1-acylglycerol-3-phosphate O-acyltransferase (AGPAT)	*plsC*	00020	*R. qingshengii*	209/245 (85%)	TAG biosynthesis	
1-acylglycerol-3-phosphate O-acyltransferase (AGPAT)	*plsC*	00059	*R. erythropolis*	297/345 (86%)	TAG biosynthesis	
Diacylglycerol acyltransferase (DGAT)	*atf*	00067	*R. qingshengii*	436/488 (89%)	TAG biosynthesis	
Glycerol-3-phosphate O-acyltransferase (GPAT)	*plsB*	00068	*R. erythropolis* PR4	640/770 (83%)	TAG biosynthesis	
1-acylglycerol-3-phosphate O-acyltransferase (AGPAT)	*plsC*	00069	*R. erythropolis* SK121	408/477 (86%)	TAG biosynthesis	
Diacylglycerol acyltransferase (DGAT)	*atf*	00162	*R. erythropolis* CCM2595	359/472 (76%)	TAG biosynthesis	
Diacylglycerol acyltransferase (DGAT)	*atf*	00170	*R. erythropolis*	343/486 (71%)	TAG biosynthesis	
1-acylglycerol-3-phosphate O-acyltransferase (AGPAT)	*plsC*	00299	*R. erythropolis* SK121	194/225 (86%)	TAG biosynthesis	
1-acylglycerol-3-phosphate O-acyltransferase (AGPAT)	*plsC*	02004	*R. erythropolis* SK121	237/251 (94%)	TAG biosynthesis	
1-acylglycerol-3-phosphate O-acyltransferase (AGPAT)	*plsC*	02005	*R. qingshengii*	179/213 (84%)	TAG biosynthesis	
Fatty acid synthase I	*fas1*	02346	*R. erythropolis*	2935/3103 (95%)	TAG biosynthesis	
Diacylglycerol acyltransferase (DGAT)	*atf*	03057	*R. erythropolis*	415/458 (91%)	TAG biosynthesis	
Diacylglycerol acyltransferase (DGAT)	*atf*	03296	*R. erythropolis*	307/464 (66%)	TAG biosynthesis	
Diacylglycerol acyltransferase (DGAT)	*atf*	03308	*R. erythropolis*	329/459 (72%)	TAG biosynthesis	
Diacylglycerol acyltransferase (DGAT)	*atf*	04005	*R. imtechensis*	355/467 (76%)	TAG biosynthesis	

Phosphatidic acid phophatase (PAP)	*pgpB*	04157	*R. erythropolis*	176/251 (70%)	TAG biosynthesis	
Phosphatidic acid phophatase (PAP)	*pgpB*	04384	*R. erythropolis*	146/178 (82%)	TAG biosynthesis	
Diacylglycerol acyltransferase (DGAT)	*atf*	04530	*R. opacus*	220/424 (52%)	TAG biosynthesis	
1-acylglycerol-3-phosphate O-acyltransferase (AGPAT)	*plsC*	05208	*R. erythropolis*	228/271 (84%)	TAG biosynthesis	
1-acylglycerol-3-phosphate O-acyltransferase (AGPAT)	*plsC*	05288	*R. qingshengii*	228/282 (81%)	TAG biosynthesis	
Diacylglycerol acyltransferase (DGAT)	*atf*	05428	*R. qingshengii*	406/469 (87%)	TAG biosynthesis	
Glycogen branching enzyme	*glgB*	02329	*R.* sp. P27	671/727 (92%)	Glycogen metabolism	70% id to GlgB from *M. tuberculosis* H37Rv
Alpha amylase	*glgE*	02330	*R. erythropolis*	609/663 (92%)	Glycogen metabolism	64% id to GlgE from *M. tuberculosis* H37Rv
Glycogen phosphorylase	*glgP*	02331	*R. erythropolis*	828/866 (96%)	Glycogen metabolism	65% id to GlgP from *M. tuberculosis* H37Rv
Glycogen synthase	*glgA*	04814	*R. erythropolis* PR4	336/386 (87%)	Glycogen metabolism	66% id to Rv1212C from *M. tuberculosis* H37Rv
Glucose-1-phosphate adenylyltransferase	*glgC*	04815	*R. erythropolis* PR4	393/398 (99%)	Glycogen metabolism	
Glycogen debranching enzyme	*treX*	05140	*R. erythropolis*	698/787 (89%)	Glycogen metabolism	78% id to TreX from *M. tuberculosis* H37Rv
Malto-oligosyl trehalose synthase	*treY*	05141	*R. erythropolis*	593/783 (76%)	Glycogen metabolism	50% id to TreY from *M. tuberculosis* H37Rv
Malto-oligosyl trehalose trehalohydrolase	*treZ*	05150	*R. erythropolis*	504/573 (88%)	Glycogen metabolism	64% id to TreZ from *M. tuberculosis* H37Rv
SAM-dependent methyltransferase		05292	*R.* sp. P27	259/281 (92%)	Glycogen metabolism	65% id to Rv3030 from *M. tuberculosis* H37Rv
1,4-alpha-glucan branching protein		05293	*R. erythropolis* PR4	474/519 (91%)	Glycogen metabolism	68% id to Rv3031 from *M. tuberculosis* H37Rv
Glycosyltransferase	*glgA*	05294	*R. erythropolis*	394/414 (95%)	Glycogen metabolism	70% id to Rv3032 from *M. tuberculosis* H37Rv
Debranching enzyme	*glgX*	05303	*R. erythropolis*	584/723 (81%)	Glycogen metabolism	47% id to GlgX from E. coli
Exopolyphosphatase	*ppx*	00072	*R. erythropolis* CCM2595	276/288 (96%)	Polyphosphate metabolism	
Polyphosphate kinase	*ppk*	02700	*R. erythropolis* PR4	679/758 (90%)	Polyphosphate metabolism	
Exopolyphosphatase	*ppx*	02851	*R. qingshengii*	272/334 (81%)	Polyphosphate metabolism	
Exopolyphosphatase	*ppx*	04676	*R. erythropolis*	272/311 (87%)	Polyphosphate metabolism	

Alkane metabolism / cytochrome p450

Enzyme	Gene	SZ00_	Best NCBI Blastp hit	Amino acid % id	Comments (reference)
Cytochrome p450 steroid MO		00187	*R. erythropolis*	414/422 (98%)	
Cytochrome p450 144		00507	*R. rhodochrous*	337/423 (80%)	
Cytochrome p450 130		00669	*R. qingshengii*	358/385 (93%)	
Cytochrome p450 144		00785	*Nocardia* BMG111209	276/421 (66%)	
Cytochrome p450 144		00835	*Frankia* EAN1pec	320/427 (75%)	

Ferredoxin		00836	*Frankia* EAN1pec	42/62 (68%)	
Cytochrome p450 144		00917	*R. erythropolis* CCM2595	271/400 (68%)	
Cytochrome p450 SU2		00923	*R. erythropolis* PR4	312/410 (76%)	
Ferredoxin		01219	*R. rhodochrous*	81/105 (77%)	
Cytochrome p450 256		01220	*R*. sp. RHA1	335/410 (82%)	
Cytochrome p450		01678	*R. rhodochrous* ATCC 21198	340/412 (83%)	
Rhodocoxin reductase		02121	*Sporichthya polymorpha*	224/404 (55%)	
Ferredoxin		02122	*Actinomycetospora chiangmaiensis*	35/61 (57%)	
Cytochrome p450 124		02123	*Streptomyces scabrisporus*	255/427 (60%)	
Cytochrome p450 116		02223	*R. equi*	661/768 (86%)	
Alkane hydroxylase	*alkB*	02689	*R. erythropolis*	335/386 (87%)	43% id to *A. borkumensis* AP1 (CAC38027) (3)
Cytochrome p450 BM3		03114	*R. triatomae*	288/447 (64%)	
Alkane hydroxylase	*alkB*	03717	*R. erythropolis*	301/377 (80%)	43% id to *P. oleovorans* (P12691.1) (4)
Cytochrome p450 Cam		03936	*R. rhodochrous*	379/448 (85%)	
Ferredoxin		03937			
Cytochrome p450 142		03954	*R. erythropolis* CCM2595	350/401 (87%)	
Cytochrome p450 BM3		03993	*R. erythropolis* PR4	197/216 (91%)	
Cytochrome p450 BM3		03994	*R. erythropolis* PR4	203/229 (89%)	
Cytochrome p450 120		04035	*R. erythropolis* PR4	415/454 (91%)	
Cytochrome p450 steroid MO		04082	*R. erythropolis* PR4	399/416 (96%)	
Cytochrome p450 steroid MO		05010	*R. erythropolis* CCM2595	371/417 (89%)	
Ferredoxin		05014	*R. triatomae*	48/64 (75%)	
Cytochrome p450		05015	*R. qingshengii*	421/452 (93%)	
Cytochrome p450 107B1		05017	*R. erythropolis* PR4	343/401 (86%)	
Rubredoxin		05444	*R. erythropolis* PR4	55/59 (93%)	
Rubredoxin		05445	*R. erythropolis* PR4	47/58 (81%)	
Alkane hydroxylase	*alkB*	05446	*R. erythropolis* PR4	362/408 (89%)	69% id to *M.tuberculosis* H37Rv (O05895) (3)
Cytochrome p450		05802	*Acidobacteriaceae bacterium* KBS 96	237/394 (60%)	
Ferredoxin reductase		06165	*Amycolatopsis* sp. ATCC 39116	219/406 (54%)	Plasmid encoded
Ferredoxin		06166	*Gordonia otitidis*	68/106 (64%)	Plasmid encoded

Cytochrome p450 terp	06167	*Gordonia otitidis*	228/402 (57%)	Plasmid encoded

Aromatic metabolism

Description	Gene	SZ00_	Best NCBI Blastp hit	Amino acid % id	query strain (reference)	query strain %id
Acetyl-CoA acyltransferase	*pcaF*	04335	*R. erythropolis* CCM2595	382/403 (95%)	*R*. sp. RHA1 (5)	81
Transcriptional regulator	*pcaR*	04336	*R. erythropolis* CCM2595	251/274 (92%)	*R*. sp. RHA1 (5)	70
3-oxoadipate eno-lactone hydrolase/4-carboxymuconolactone decarboxylase	*pcaL*	04337	*R. erythropolis* CCM2595	360/392 (92%)	*R*. sp. RHA1 (5)	63
3-carboxy-cis, cis-muconate cycloisomerase	*pcaB*	04338	*R. erythropolis*	396/442 (90%)	*R*. sp. RHA1 (5)	61
Protocatechuate dioxygenase, α-subunit	*pcaG*	04339	*R. erythropolis*	201/223 (90%)	*R*. sp. RHA1 (5)	64
Protocatechuate dioxygenase, β-subunit	*pcaH*	04340	*R. erythropolis* CCM2595	233/239 (97%)	*R*. sp. RHA1 (5)	72
3-oxoadipate CoA-transferase, α-subunit	*pcaI*	04341	*R. erythropolis*	247/249 (99%)	*R*. sp. RHA1 (5)	80
3-oxoadipate CoA-transferase, β-subunit	*pcaJ*	04342	*R. erythropolis* PR4	206/210 (98%)	*R*. sp. RHA1 (5)	79
Muconolactone delta-isomerase	*catC*	01309	*R. erythropolis*	90/93 (97%)	*R. erythropolis* CCM2595 (6)	95
Muconate cycloisomerase	*catB*	01310	*R. erythropolis* PR4	366/373 (98%)	*R. erythropolis* CCM2595 (6)	98
Catechol 1,2-dioxygenase	*catA*	01311	*R. erythropolis*	273/279 (98%)	*R. erythropolis* CCM2595 (6)	97
Transcriptional regulator	*catR*	01312	*R. erythropolis*	251/256 (98%)	*R. erythropolis* CCM2595 (7)	96
Transcriptional regulator	*araC*	01313	*R. qingshengii*	304/311 (98%)	*R*. sp. RHA1	62
Phenol hydroxylase reductase	*pheA2*	01314	*R. qingshengii*	187/189 (99%)	*R. erythropolis* UPV-1 (8)	98
Phenol hydroxylase	*pheA1*	01315	*R. erythropolis*	541/542 (99%)	*R. erythropolis* UPV-1 (8)	99
Benzoate 1,2-dioxygenase, α-subunit	*benA*	01316	*R. erythropolis* SK121	447/464 (96%)	*R*. sp. RHA1 (9)	77
Benzoate 1,2-dioxygenase, β-subunit	*benB*	01317	*R. erythropolis* SK121	157/169 (93%)	*R*. sp. RHA1 (9)	85
Benzoate 1,2-dioxygenase, reductase	*benC*	01318	*R. erythropolis*	466/510 (91%)	*R*. sp. RHA1 (9)	76
Benzoate diol dehydrogenase	*benD*	01319	*R. erythropolis*	237/260 (91%)	*R*. sp. RHA1 (9)	76
Transporter	*benK*	01320	*R. qingshengii*	423/460 (92%)	*R*. sp. RHA1 (9)	78
4-hydroxybenzoate hydroxylase	*pobA2*	00624	*R. triatomae*	308/393 (78%)	*P. aeruginosa* PAO1 (10)	45
4-hydroxybenzoate hydroxylase	*pobA1*	01101	*Gordonia* sp. NB4-1Y	346/400 (87%)	*P. aeruginosa* PAO1 (10)	48
3-hydroxybenzoate hydroxylase	*narX*	01673	*Nocardia* sp. 348MFTsu5.1	357/412 (87%)	*R*. NCIMB 12038 (11)	51
Mycothiol dependent malelypyruvate isomerase	*narL*	01674	*Nocardia* sp. 348MFTsu5.2	162/229 (71%)	*C. glutamicum* ATCC 13032 (12)	43
Fumarylpyruvate hydrolase	*narK*	01675	*Nocardia* sp. 348MFTsu5.3	232/278 (83%)	*R*. NCIMB 12038 (11)	48
Gentisate dioxygenase	*narI*	01676	*Nocardia* sp. 348MFTsu5.4	336/378 (89%)	*R*. NCIMB 12038 (11)	61

Transcriptional regulator IcLR family	*narR*	01677	*Nocardia* sp. 348MFTsu5.5	205/255 (80%)	*R*. NCIMB 12038 (11)	39
Transcriptional regulator IclR family	*padR*	03580	*Nocardioides insulae*	185/251 (74%)	*R*. sp. RHA1 (5)	56
3,4-dihydroxyphthalate decarboxylase	*padC*	03592	*Gordonia namibiensis*	168/233 (72%)	*R*. sp. RHA1 (5)	55
Phthalate 3,4-dioxygenase ferredoxin reductase	*padAd*	03593	*Gordonia namibiensis*	214/378 (57%)	*R*. sp. RHA1 (5)	52
Ferredoxin	*padAc*	03594	*Saccharomonospora azurea*	42/65 (65%)	*R*. sp. RHA1 (5)	65
Phthalate 3,4-dihydrodiol hydrogenase	*padB*	03595	*Gordonia namibiensis*	193/260 (74%)	*R*. sp. RHA1 (5)	70
Hypothetical		03596	*Nocardioides insulae*	72/97 (74%)	*R*. sp. RHA1 (5)	51
Phthalate 3,4-dioxygenase β- subunit	*padAb*	03597	*Gordonia namibiensis*	150/195 (77%)	*R*. sp. RHA1 (5)	64
Phthalate 3,4-dioxygenase α-subunit	*padAa*	03598	*Gordonia namibiensis*	406/464 (88%)	*R*. sp. RHA1 (5)	77

Replication-associated genes

Description	Replicon	Gene	SZ00_	Best NCBI Blastp hit	amino acid % id
DNA gyrase	Chromosome	*gyrA*	01813	*R. erythropolis* PR4	814/843(97%)
DNA gyrase	Chromosome	*gyrB*	01812	*R. imtechensis*	616/680(91%)
Recombination protein	Chromosome	*recF*	01806	*R. erythropolis*	366/396(92%)
DNA polymerase subunit	Chromosome	*dnaN*	01805	*R. erythropolis* PR4	373/394(95%)
Replication initiation protein	Chromosome	*dnaA*	01804	*R. erythropolis* PR4	500/520(96%)
RNase P component	Chromosome	*rnpA*	01803	*R. erythropolis*	115/126(91%)
Partitioning protein	Chromosome	*parA*	01795	*R. qingshengii*	307/328(94%)
Partitioning protein	Chromosome	*parB*	01794	*R. qingshengii*	242/260(93%)
Replication protein	Plasmid	*rep*	06269	*R. opacus*	211/440(48%)
Partitioning protein	Plasmid	*parA*	06266	*R. qingshengii*	310/361(86%)
Toxin/antitoxin component	Plasmid	*relB*	06060	*R*. sp. EsD8	78/94(83%)
Toxin/antitoxin component	Plasmid	*relE*	06059	*R*. sp. EsD8	73/93(78%)

References: (1), Eikmanns et al., (1989); (2), Koffas et al., (1998); (3), Smits et al., (2002); (4), Kok et al., (1989); (5), Patrauchan et al., (2005); (6), Knoppová et al., (2007); (7), Vesely et al., (2007); (8), Saa et al., (2010); (9), Kitagawa et al., (2001); (10), Entsch et al., (1988); (11), Liu et al., (2011); (12), Feng et al., (2006).

Table S3. Frequency distribution of gene transcription. Transcript abundance is shown as % of total RPKM for each sample and timepoint. Data show the mean of three replicates or 15 replicates (T0) except that six samples in total were excluded as described in *Experimental procedures*.

	T0	T1	T1	T1	T1	T1	T2	T2	T2	T2	T2
RPKM	%	EPI	Glu	Iso	N-S	Succ	EPI	Glu	Iso	N-S	Succ
<2.5	8.06	7.55	6.36	9.07	6.69	14.39	16.03	7.74	10.49	8.91	15.76
2.5-12.5	14.59	15.14	16.22	19.09	12.79	22.25	23.17	17.11	18.68	19.37	21.49
12.5-62.5	35.63	39.78	33.99	32.26	39.18	26.78	33.64	31.68	32.38	32.68	26.76
62.5-315	31.17	28.20	32.27	29.19	31.11	25.19	20.91	31.92	27.93	28.58	25.02
315-1575	9.41	7.97	10.05	9.18	9.07	9.76	5.30	10.46	9.18	9.21	9.28
1575-7875	1.07	1.24	1.04	1.13	1.09	1.56	0.64	1.02	1.26	1.17	1.63
>7875	0.07	0.12	0.08	0.08	0.07	0.06	0.30	0.07	0.08	0.08	0.06

	T3	T3	T3	T3	T3	T4	T4	T4	T4	T4	T5	T5	T5	T5	T5
RPKM	EPI	Glu	Iso	NS	Succ	EPI	Glu	Iso	NS	Succ	EPI	Glu	Iso	NS	Succ
<2.5	13.63	4.45	11.22	9.04	10.46	9.79	5.98	0.29	6.09	1.80	10.48	8.77	12.03	0.03	7.83
2.5-12.5	26.54	18.89	18.56	20.10	25.22	18.85	17.37	9.93	23.14	16.17	22.93	11.43	21.00	0.11	11.11
12.5-62.5	34.01	33.19	31.93	32.78	28.51	45.15	33.75	51.20	35.82	39.18	35.50	43.22	36.03	24.62	25.02
62.5-315	19.93	32.09	27.87	27.69	24.88	20.55	31.68	29.26	25.19	33.51	22.55	28.79	23.51	69.05	48.81
315-1575	4.89	10.32	9.11	9.12	9.14	4.70	9.95	8.14	8.46	8.27	7.13	6.64	6.37	5.48	6.28
1575-7875	0.68	0.98	1.24	1.20	1.74	0.66	1.19	1.10	1.21	0.99	1.31	1.09	0.80	0.67	0.88
>7875	0.31	0.09	0.07	0.08	0.04	0.30	0.07	0.08	0.08	0.08	0.09	0.06	0.26	0.04	0.06

Supplementary Figures

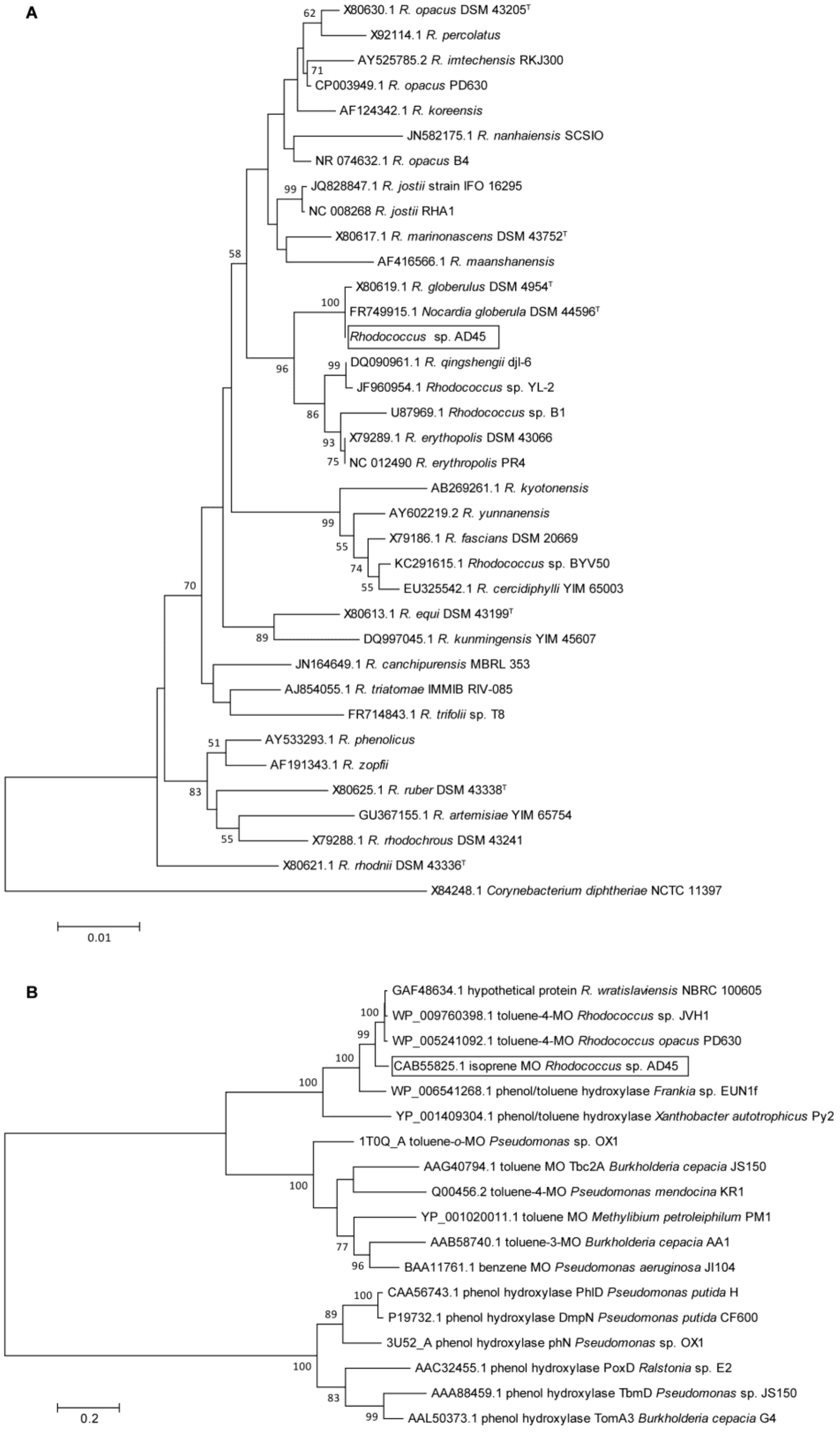

Fig. S1. (A) 16S rRNA gene-based phylogenetic relationship between *Rhodococcus* sp. AD45 (shown boxed) and other representative strains. The tree, drawn using the neighbour joining method, was constructed using MEGA6 (Tamura et al., 2013). All positions containing gaps and missing data were eliminated. There were a total of 1,321 nucleotide positions in the final dataset. Bootstrap values (1000 replications) greater than 50% are shown at the nodes. The scale bar shows nucleotide substitutions per site. (B) Un-rooted phylogenetic tree, based on amino acid sequences, relating the *Rhodococcus* sp. AD45 isoprene monooxygenase hydroxylase α-subunit with other representative enzymes. The tree was constructed using the maximum likelihood method in Mega 6 (Tamura et al., 2013). All positions containing gaps and missing data were eliminated. There were a total of 481 positions in the final dataset. Bootstrap values (500 replications) greater than 75% are shown at the nodes. The scale bar shows amino acid substitutions per site.

A

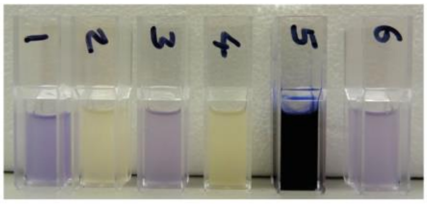

B

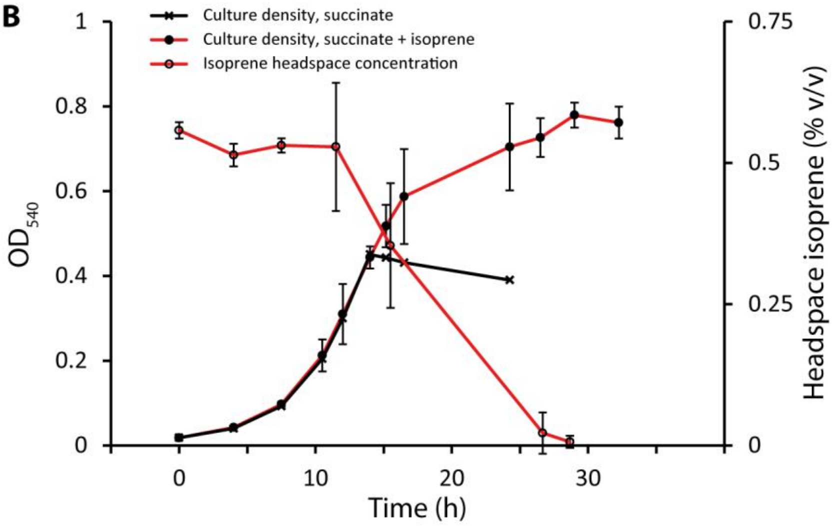

Fig. S2. (A) Epoxide forming ability of cells grown on isoprene (cuvettes 1, 3, 6) or succinate (cuvettes 2, 4) when incubated with hexene. The positive control, containing cells incubated with epoxyhexane, is shown in cuvette 5. Blue colouration indicates the presence of epoxide. (B) Growth of *Rhodococcus* sp. AD45 on succinate or succinate plus isoprene. Isoprene headspace concentration (when grown on succinate plus isoprene) is shown on the secondary (right) axis. Data show the mean ± SD of three replicates.

A

DnaA box sequences

Consensus from Moriya et al., (1985):	TTAT(C/A)CACA
Rhodococcus sp. AD45 sequences:	TTATCCACA CAG TTATCCCCA<br>GTTTCCACA CAA TTATCCACA

parS sequences

Consensus from Livny et al., (2007):	TGTTTCACGTGAAACA
Rhodococcus sp. AD45 sequences:	GGTTTCACGTGAAACG<br>GGTTTCACGTGAAACG<br>TGTTTCACGTGAAACC<br>TGTTTCACGTGGAACA<br>TGTTTCACGTGAAACA

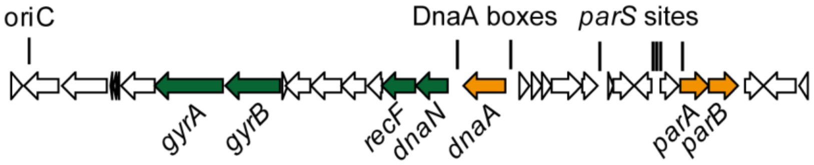

B

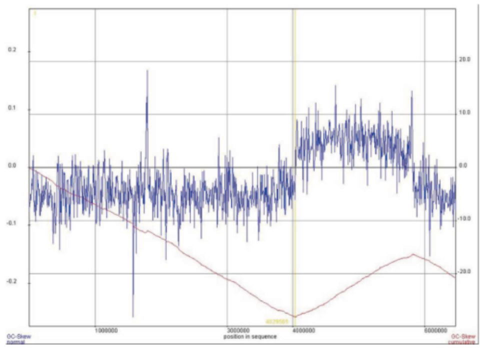

Fig. S3. Replication region of *Rhodococcus* sp. AD45. (A) The genome in the region of the putative replication origin, showing DnaA box and *parS* sequences. The consensus sequences from *Bacillus subtilis* (Moriya et al., 1985) and Gram-positive bacteria (Livny et al., 2007) are shown. (B) Guanine-cytosine (GC) asymmetry calculated using the web-based server GenSkew (http://genskew.csb.univie.ac.at/).

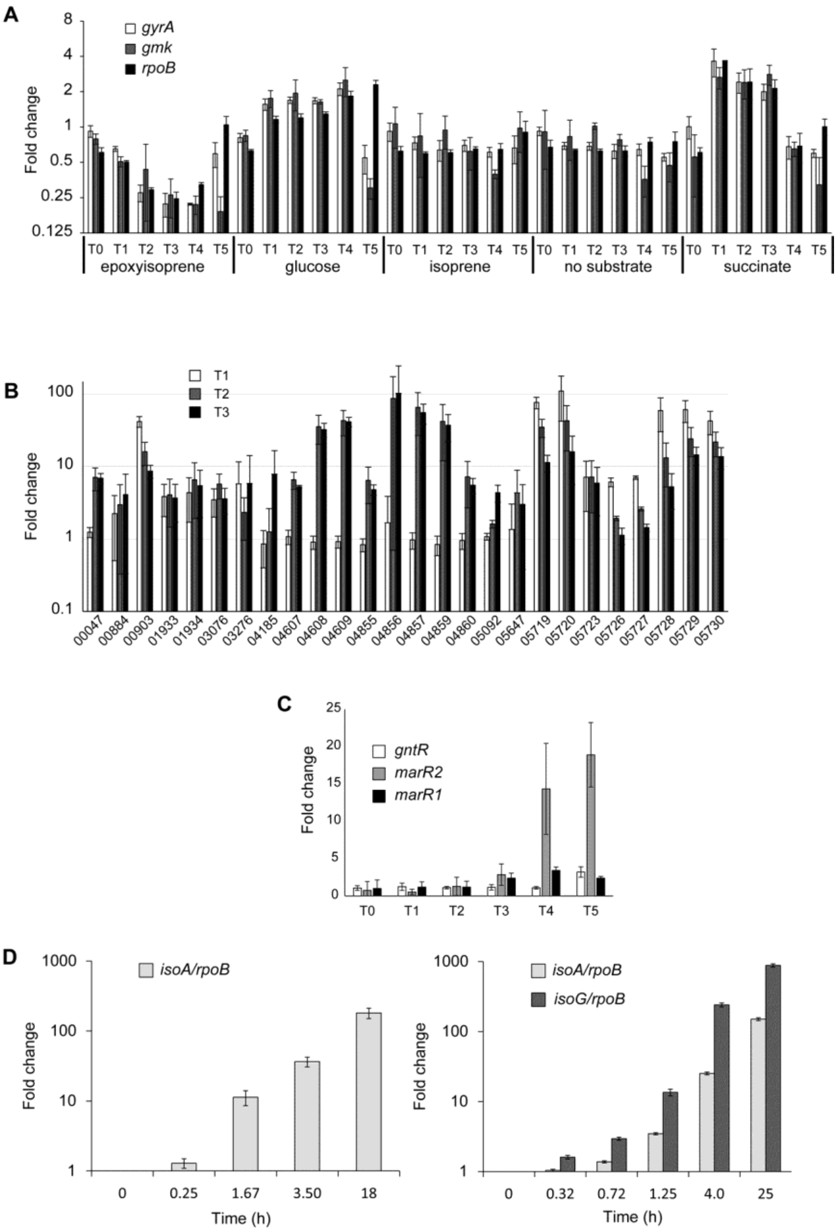

Fig. S4. (A) Transcripts of *rpoB*, *gyrA* and *gmk*, encoding the β-subunit of RNA polymerase, the α-subunit of DNA gyrase and guanylate kinase, respectively, compared over five treatments and six time-points. The bar chart shows expression level (transcript abundance, RPKM), normalised to the mean level across all conditions for each gene. Data show the mean of three replicates ± SD except for six samples that were excluded as described in *Experimental procedures*. T0 – T5 refer to the sampling time-points (time zero to 25 h) as described in the text. (B) Upregulation of genes, additional to the isoprene cluster SZ00_06080 – SZ00_06106, in epoxyisoprene-induced cells at time-points T1, T2, T3, compared with T0. All genes upregulated by at least fourfold in any of time-points T1 – T3, compared with T0, are shown. Data show the mean fold-change of three replicates ± SD. SZ00_05719 - SZ00_05730 show homology to sequences possibly involved in amino alcohol metabolism (Nagy et al., 1995). SZ00_04855 - SZ00_04859 comprise a putative enterobactin exporter, peroxidase, multidrug resistance protein and hypothetical proteins and SZ00_04607 - SZ00_04609 are annotated as copper transport proteins. None of these genes, however, was also upregulated (>fourfold) in comparison with non-induced (no-substrate) and succinate-induced samples at the same time-points. (C) Upregulation of three regulatory genes from the isoprene cluster showing the change in abundance (fold-change) from T0 to T5 in isoprene-induced samples in comparison with no-substrate controls at the same time-points. Data show the mean of three replicates ± SD, except T0, 15 replicates. (D) Upregulation of *isoA* (left) and *isoA* and *isoG* (right), in succinate-grown cells, induced by isoprene, quantified by RT-qPCR. The data are normalised to *rpoB* as reference gene, expressed relative to time-point zero, prior to induction, and show the mean ± SD of three technical replicates from the two independent experiments, quantifying *isoA* (left) or both *isoA* and *isoG* (right).

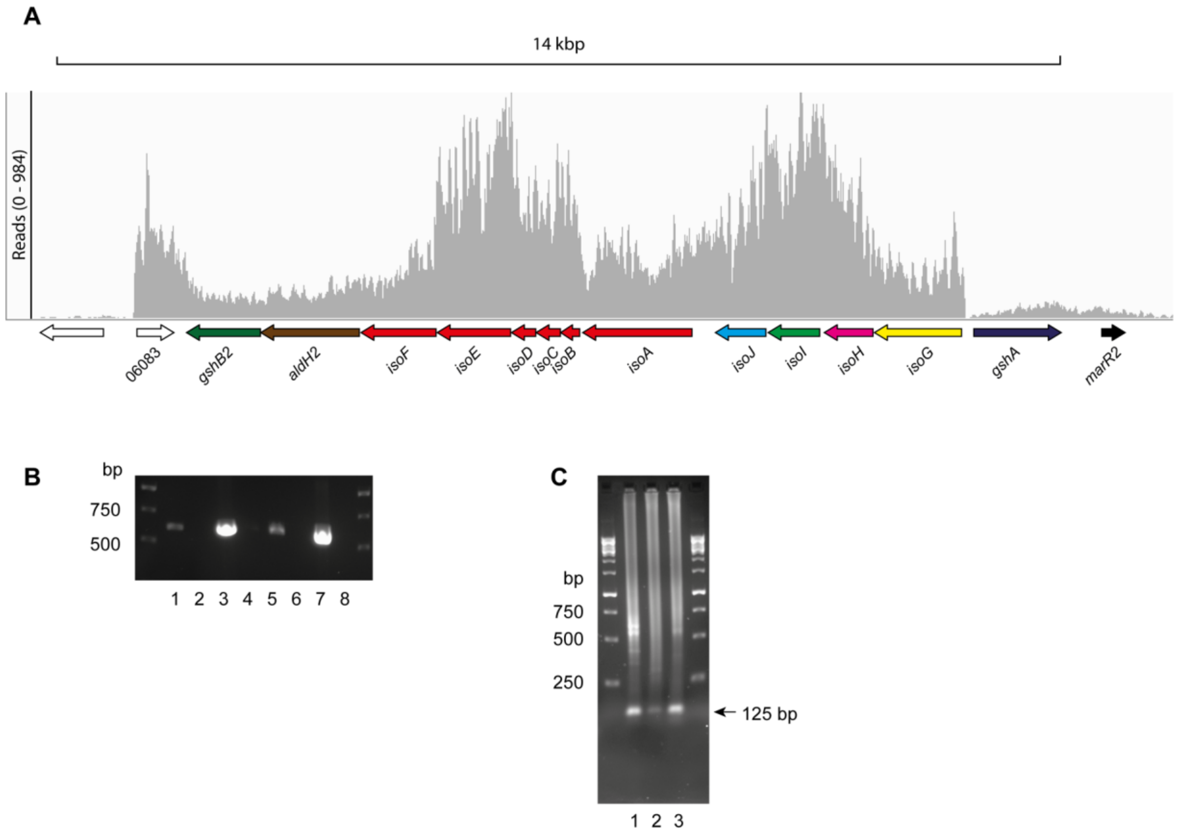

Fig. S5. (A) Transcript reads mapped to the isoprene cluster region of the *Rhodococcus* sp. AD45 genome, displayed in IGV (Thorvaldsdóttir et al., 2013). The trace, indicating read abundance, shows replicate 2 induced with isoprene at T5, and is representative of three replicates. The y-axis shows read coverage at each nucleotide position. The ORFs are shown below. The trace indicates that transcription start sites are located upstream of *isoG, gshA* and SZ00_06083. Additionally, no termination of transcription is apparent in the *isoJ – isoA* intergene space. (B) PCR using primers spanning the *isoJ-isoA* intergene region using a cDNA template. Complementary DNA was synthesised from RNA extracted from cells grown on succinate (lane 1), isoprene (3) or epoxyisoprene (5). Lane 7 used a DNA template. Lanes 2, 4, 6 omitted reverse transcriptase from the cDNA synthesis reactions. Lane 8, no template control. Expected product size, 617 bp. The amplification of cDNA from all three growth conditions demonstrates the continuity of transcription across this region. (C) 5' RACE was used with RNA extracted from cells grown on epoxyisoprene (lane 1), isoprene (2) or succinate (3). The 125 bp band was cloned and sequenced, indicating a transcriptional start site approximately 75 bp 5' of the *isoG* start codon.

References

Eikmanns, B., Follettie, M., Griot, M., and Sinskey, A. (1989) The phosphoenolpyruvate carboxylase gene of *Corynebacterium glutamicum*: molecular cloning, nucleotide sequence, and expression. *Mol Gen Genet* **218**: 330-339.

Entsch, B., Nan, Y., Weaich, K., and Scott, K.F. (1988) Sequence and organization of *pobA*, the gene coding for p-hydroxybenzoate hydroxylase, an inducible enzyme from *Pseudomonas aeruginosa*. *Gene* **71**: 279-291.

Feng, J., Che, Y., Milse, J., Yin, Y.J., Liu, L., Ruckert, C. et al. (2006) The gene *ncgl2918* encodes a novel maleylpyruvate isomerase that needs mycothiol as cofactor and links mycothiol biosynthesis and gentisate assimilation in *Corynebacterium glutamicum*. *J Biol Chem* **281**: 10778-10785.

Kitagawa, W., Miyauchi, K., Masai, E., and Fukuda, M. (2001) Cloning and characterization of benzoate catabolic genes in the Gram-positive polychlorinated biphenyl degrader *Rhodococcus* sp. strain RHA1. *J Bacteriol* **183**: 6598-6606.

Knoppová, M., Phensaijai, M., Veselý, M., Zemanová, M., Nešvera, J., and Pátek, M. (2007) Plasmid vectors for testing in vivo promoter activities in *Corynebacterium glutamicum* and *Rhodococcus erythropolis*. *Curr Microbiol* **55**: 234-239.

Koffas, M.A.G., Ramamoorthi, R., Pine, W.A., Sinskey, A.J., and Stephanopoulos, G. (1998) Sequence of the *Corynebacterium glutamicum* pyruvate carboxylase gene. *Appl Microbiol Biotechnol* **50**: 346-352.

Kok, M., Oldenhuis, R., van der Linden, M.P., Raatjes, P., Kingma, J., van Lelyveld, P.H., and Witholt, B. (1989) The *Pseudomonas oleovorans* alkane hydroxylase gene. Sequence and expression. *J Biol Chem* **264**: 5435-5441.

Liu, T.-T., Xu, Y., Liu, H., Luo, S., Yin, Y.-J., Liu, S.-J., and Zhou, N.-Y. (2011) Functional characterization of a gene cluster involved in gentisate catabolism in *Rhodococcus* sp. strain NCIMB 12038. *Appl Microbiol Biotechnol* **90**: 671-678.

Livny, J., Yamaichi, Y., and Waldor, M.K. (2007) Distribution of centromere-like *parS* sites in bacteria: insights from comparative genomics. *J Bacteriol* **189**: 8693-8703.

Moriya, S., Ogasawara, N., and Yoshikawa, H. (1985) Structure and function of the region of the replication origin of the *Bacillus subtilis* chromosome. III. Nucleotide sequence of some 10,000 base pairs in the origin region. *Nucleic Acids Res* **13**: 2251-2265.
Nagy, I., Schoofs, G., Compernolle, F., Proost, P., Vanderleyden, J., and de Mot, R. (1995) Degradation of the thiocarbamate herbicide EPTC (S-ethyl dipropylcarbamothioate) and biosafening by *Rhodococcus* sp. strain NI86/21 involve an inducible cytochrome P-450 system and aldehyde dehydrogenase. *J Bacteriol* **177**: 676-687.
Patrauchan, M.A., Florizone, C., Dosanjh, M., Mohn, W.W., Davies, J., and Eltis, L.D. (2005) Catabolism of benzoate and phthalate in *Rhodococcus* sp. strain RHA1: redundancies and convergence. *J Bacteriol* **187**: 4050-4063.
Saa, L., Jaureguibeitia, A., Largo, E., Llama, M., and Serra, J. (2010) Cloning, purification and characterization of two components of phenol hydroxylase from *Rhodococcus erythropolis* UPV-1. *Appl Microbiol Biotechnol* **86**: 201-211.
Smits, T.H.M., Balada, S.B., Witholt, B., and van Beilen, J.B. (2002) Functional analysis of alkane hydroxylases from Gram-negative and Gram-positive bacteria. *J Bacteriol* **184**: 1733-1742.
Tamura, K., Stecher, G., Peterson, D., Filipski, A., and Kumar, S. (2013) MEGA6: Molecular Evolutionary Genetics Analysis version 6.0. *Mol Biol Evol*.
Thorvaldsdóttir, H., Robinson, J.T., and Mesirov, J.P. (2013) Integrative Genomics Viewer (IGV): high-performance genomics data visualization and exploration. *Brief Bioinform* **14**: 178-192.
Vesely, M., Knoppova, M., Nesvera, J., and Patek, M. (2007) Analysis of *catRABC* operon for catechol degradation from phenol-degrading *Rhodococcus erythropolis*. *Appl Microbiol Biotechnol* **76**: 159-168.
